# Scaling Relation between the Reduction Potential of Copper Catalysts and the Turnover Frequency for the Oxygen and Hydrogen Peroxide Reduction Reactions

**DOI:** 10.1021/acs.inorgchem.3c02939

**Published:** 2023-11-17

**Authors:** Michiel Langerman, Phebe H. van Langevelde, Johannes J. van de Vijver, Maxime A. Siegler, Dennis G. H. Hetterscheid

**Affiliations:** † Leiden Institute of Chemistry, Leiden University, Einsteinweg 55, 2300 RA Leiden, The Netherlands; ‡ Department of Chemistry, Johns Hopkins University, 3400 North Charles St., Baltimore, Maryland 21218, United States

## Abstract

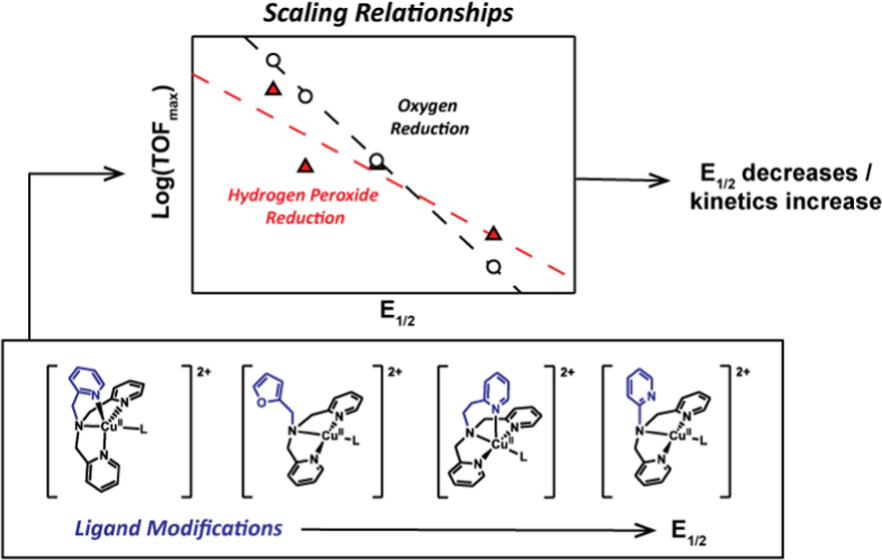

Changes in the electronic structure of copper complexes can have a remarkable impact on the catalytic rates, selectivity, and overpotential of electrocatalytic reactions. We have investigated the effect of the half-wave potential (*E*_1/2_) of the Cu^II^/Cu^I^ redox couples of four copper complexes with different pyridylalkylamine ligands. A linear relationship was found between *E*_1/2_ of the catalysts and the logarithm of the maximum rate constant of the reduction of O_2_ and H_2_O_2_. Computed binding constants of the binding of O_2_ to Cu^I^, which is the rate-determining step of the oxygen reduction reaction, also correlate with *E*_1/2_. Higher catalytic rates were found for catalysts with more negative *E*_1/2_ values, while catalytic reactions with lower overpotentials were found for complexes with more positive *E*_1/2_ values. The reduction of O_2_ is more strongly affected by the *E*_1/2_ than the H_2_O_2_ rates, resulting in that the faster catalysts are prone to accumulate peroxide, while the catalysts operating with a low overpotential are set up to accommodate the 4-electron reduction to water. This work shows that the *E*_1/2_ is an important descriptor in copper-mediated O_2_ reduction and that producing hydrogen peroxide selectively close to its equilibrium potential at 0.68 V vs reversible hydrogen electrode (RHE) may not be easy.

## Abstract 2

The *E*_1/2_ of the Cu^I^/Cu^II^ redox couple correlates with the binding energy of O_2_ to Cu(I), the log(TOF_max_) of the O_2_ reduction reaction, and the log(TOF_max_) of the H_2_O_2_ reduction reaction. It is therefore an important descriptor for both the oxygen reduction activity and selectivity.

## Introduction

**Scheme 1:**
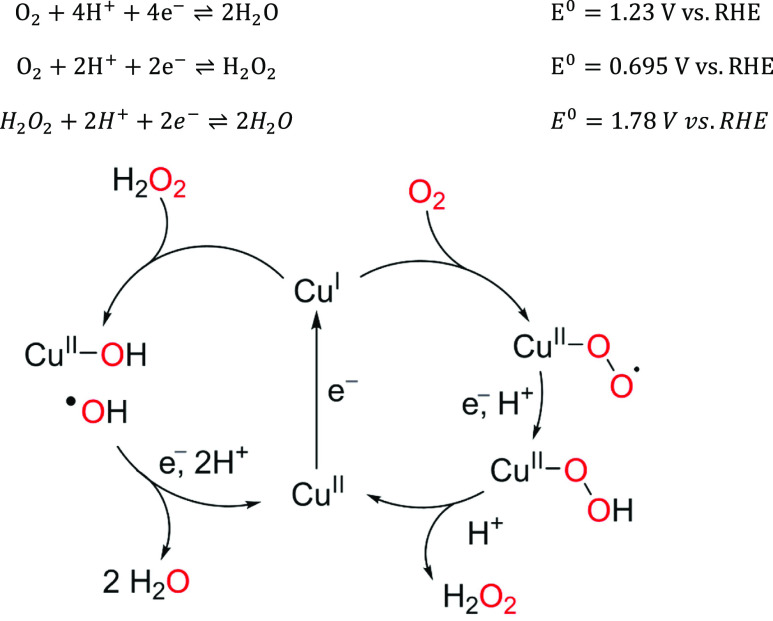
Standard Electrode Potentials of the Different Catalytic Reactions Involved in the ORR, and Postulated Mechanism for the ORR and Hydrogen Peroxide Reduction Reaction (HPRR) Mediated by Cu-tmpa

The electrochemical oxygen reduction reaction (ORR) can either result in the 4-electron reaction product (H_2_O) or the 2-electron reaction product (H_2_O_2_), both involving different standard equilibrium potentials for the respective reactions involved, as shown in Scheme 1. Additionally, the 4-electron pathway may proceed via H_2_O_2_ as an intermediate as a result of two consecutive 2H^+^/2e^–^ reaction steps. Both the 4-electron reduction of dioxygen (O_2_) to water and 2-electron reduction to H_2_O_2_ are important reactions in relation to their application in fuel cell technology and the use of H_2_O_2_ as a powerful oxidant and potential energy carrier.^1−7^

Inspired by enzymes such as copper-based monooxygenases and oxidases, the chemistry of copper complexes with O_2_ has been widely explored.^8−12^ Many potential catalytic intermediates have been spectroscopically identified, or even isolated, and their reactivity toward various reactants has been thoroughly studied by the bioinorganic chemistry community.^13−17^ A smaller amount of copper complexes have been successfully studied for the electrochemical reduction of dioxygen, in which some of the more elegant studies showed a good correlation between biology, bioinorganic model systems, and electrochemical behavior.^18^ However, many of the studies directed toward the ORR output have been carried out with less well-defined heterogenized and amorphous samples,^19,20^ and clear design principles regarding fast and selective copper-based electrocatalysts for the ORR have not yet been established.^21^

Recently, we showed that the tetradentate copper complex [Cu(tmpa)(L)]^2+^ (Cu-tmpa) (tmpa = tris(2-pyridylmethyl)amine), (L = solvent), has very high reaction rates for the electrochemical ORR.^22−24^ It was shown that a full reduction of dioxygen to water takes place via a stepwise process with H_2_O_2_ as a detectable intermediate. Both the partial reduction of O_2_ to water and the reduction of H_2_O_2_ catalyzed by Cu-tmpa demonstrated high catalytic rate constants, with only a small difference in onset potential between the 2-electron ORR and the hydrogen peroxide reduction reaction (HPRR).^22,23^ This resulted in a small potential window, where H_2_O_2_ is the primary product during catalysis. Additionally, the fast catalytic rates for both reactions come at the cost of a significant overpotential. In order to reduce the overpotential and steer the selectivity toward either the full 4-electron or 2-electron reduction of dioxygen, a better fundamental understanding is necessary between the (electronic) structure of the copper catalyst and the catalytic activity for the ORR and HPRR.

**Scheme 2:**
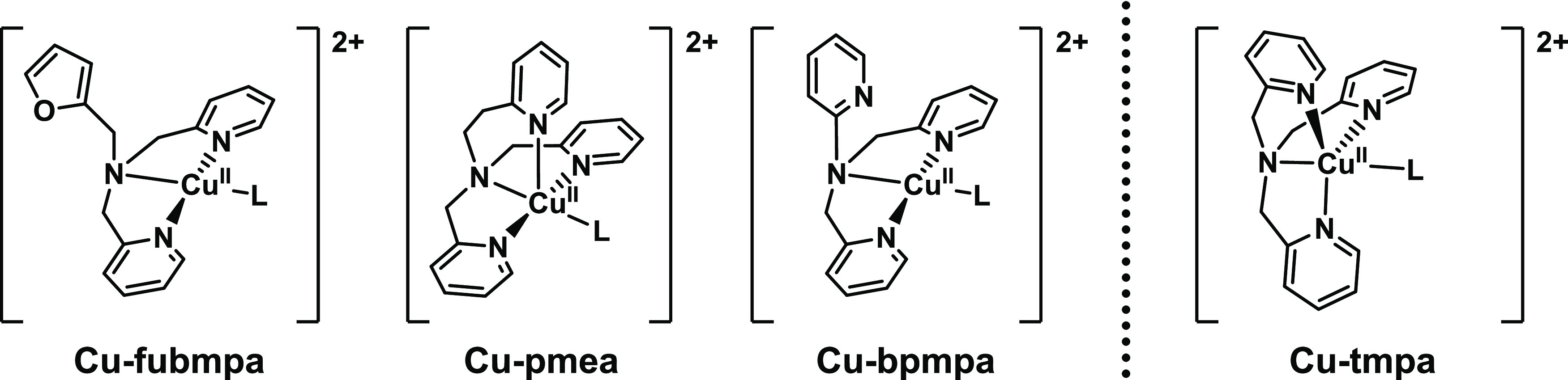
Overview of the Structures of the Three Different Copper(II) Complexes Investigated in This Work in Addition to Cu-tmpa

While a correlation between the catalytic ORR activity and the electronic structure of these Cu complexes has not been addressed in any form, the effect of ligand denticity and flexibility on the geometry and electronic structure of copper complexes has been a subject of intense study.^25−33^ A significant library of different ligand modifications has been investigated for copper complexes based on the tetradentate pyridine ligand scaffold of Cu-tmpa.^34−36^ In light of structure–activity correlations in Cu-catalyzed ORR, the half-wave potential (*E*_1/2_) is a particularly interesting parameter, given that the reduction of Cu from the +II to the +I oxidation state is potential-determining for the more competent catalytic systems.^22^ We have therefore selected a number of catalysts (Scheme 2) from the literature and some of our own previously unpublished work to investigate the relationship between *E*_1/2_ and the catalytic performance in the ORR and HPRR by employing voltammetry, rotating ring-disk experiments, and density functional theory (DFT) calculations.

## Results and Discussion

### Selection of Catalysts

In addition to the previously reported Cu-tmpa system,^22,23^ we selected three different mononuclear copper complexes, as shown in Scheme 2. In two of these, [Cu(pmea)(L)]^2+^ (Cu-pmea; pmea = bis[(2-pyridyl)methyl]-2-(2-pyridyl)ethylamine) and [Cu(bpmpa)(L)]^2+^ (Cu-bpmpa; bpmpa = bis[(2-pyridyl)methyl]-2-pyridylamine), the distance between the central tertiary amine and one of the pyridine arms was varied by changing methylene to an ethylene spacer (Cu-pmea) or removing it altogether, resulting in an aminopyridine moiety (Cu-bpmpa). A crystal structure of [Cu(bpmpa)(Cl)]ClO_4_ shows that the pyridine N of the aminopyridine does not coordinate with the copper center.^28^ This noncoordinated pyridine moiety could interact with protons present in intermediate species during the ORR and HPRR reactions. Proton shuttles in the second coordination sphere have led to a significant increase in turnover frequency (TOF) for many catalytic systems.^37,38^ Given that no H^+^ transfer is involved in the rate-determining step of the Cu-mediated ORR, we do not anticipate a significant effect on the catalytic rate here.^22,23^ The third complex, [Cu(fubmpa)(H_2_O)(OTf)_2_] (Cu-fubmpa; fubmpa = *N*-(furan-2-ylmethyl)-*N*-[bis(2-pyridyl)methyl]amine), was designed as an analogue of the copper complex [Cu(bmpa)(L)]^2+^ (bmpa = bis(2-pyridylmethyl)amine),^39^ by introduction of the noncoordinating furanyl moiety while maintaining the nature of the central tertiary amine. We have selected these complexes because their *E*_1/2_ values range between 0.2 and 0.5 V vs reversible hydrogen electrode (RHE), and, as far as we could observe, no limitations in electron transfer rates occurred within this selected series of tmpa modifications (see below), as opposed to our previous observations in the case of rigid terpyridine catalysts.^39^

### Synthesis

**Figure 1:**
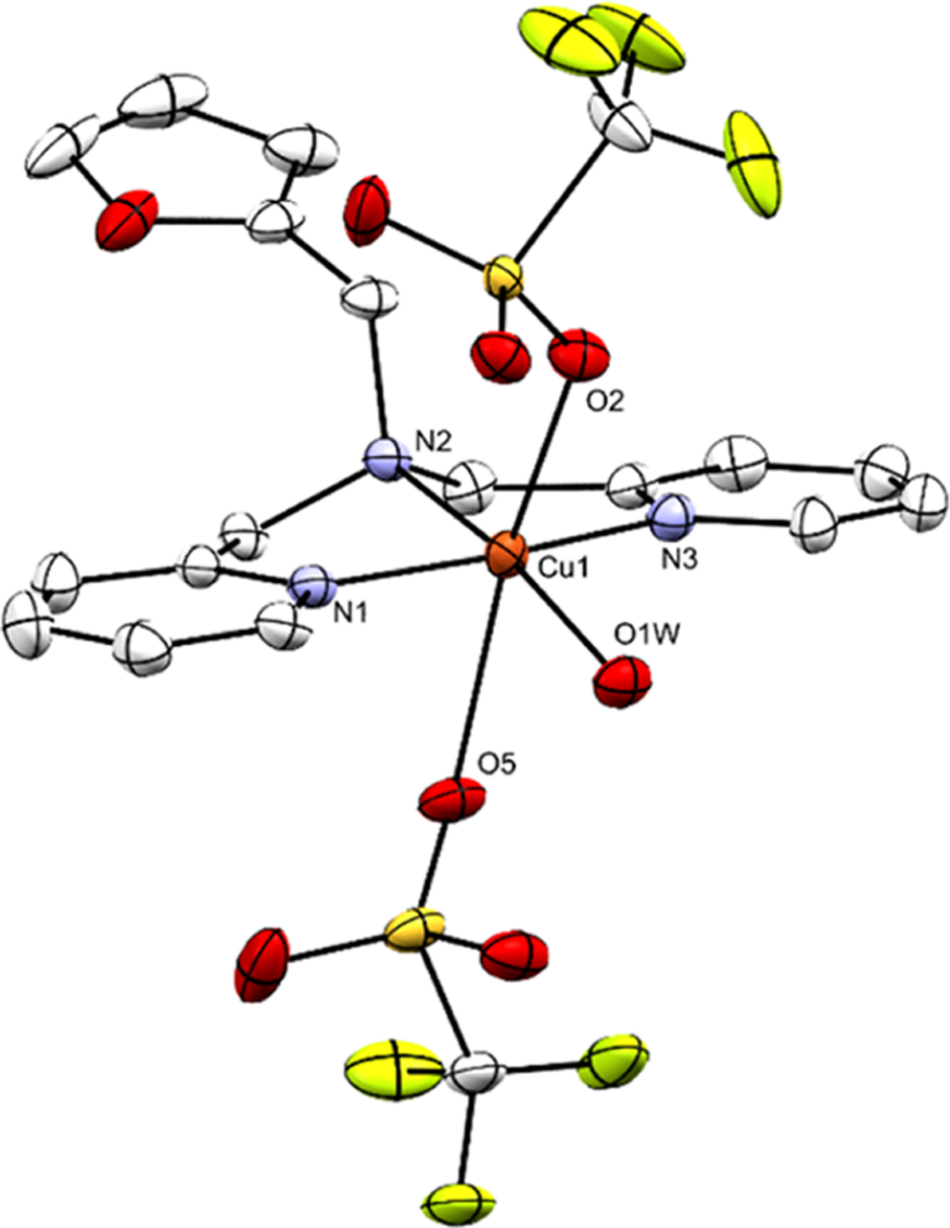
Displacement ellipsoid plot (50% probability level) of Cu-fubmpa at 110(2) K. The H atoms, disorder, and lattice water solvent molecules are omitted for clarity. Hydrogen bond interactions of O1W with several lattice water solvent molecules are shown in Figure S7.

The polypyridyl ligands bis[(2-pyridyl)methyl]-2-(2-pyridyl)ethylamine (pmea) and bis[(2-pyridyl)methyl]-2-pyridylamine (bpmpa) have been previously reported and were synthesized in a one-step reaction via reductive amination and nucleophilic substitution (S_N_2), respectively.^28,40^ The ligand *N*-(furan-2-ylmethyl)-*N*-[bis(2-pyridyl)methyl]amine (fubmpa) was synthesized from commercially available furan-2-ylmethanamine and 2-pyridinecarboxaldehyde via a reductive amination in a one-step reaction. Following purification by column chromatography, fubmpa was characterized by ^1^H NMR, ^13^C NMR, and electrospray ionization mass spectrometry (ESI MS). The copper complexes, [Cu(pmea)(CH_3_CN)](OTf)_2_ and [Cu(bpmpa)(CH_3_CN)](OTf)_2_, were synthesized by mixing the respective ligand with Cu(OTf)_2_ in a 1:1 ratio in dry CH_3_CN under an inert atmosphere, and characterization was performed by ESI MS and elemental analysis (see the Experimental Section). The copper complex [Cu(fubmpa)(H_2_O)(OTf)_2_] was synthesized by mixing fubmpa with Cu(OTf)_2_ in a 1:1 ratio in CH_3_CN. The resulting complex was purified by crystallizing the complex twice from CH_3_CN by the addition of diethyl ether. Characterization of Cu-fubmpa was done by elemental analysis, single-crystal X-ray crystallography, and UV–vis spectroscopy. The single crystals suitable for X-ray structure determination were obtained via liquid–liquid diffusion in an NMR tube, with Cu-fubmpa dissolved in chloroform and layered with diethyl ether. A projection of the structure is shown in Figure 1. In the crystal structure, the top axial OTf^–^ ligand has a Cu–O bond distance of 2.3749(15) Å. However, the Cu1–O5 distance between the copper center and the second triflate is 2.6646(16) Å. This is on the long side for an axial Cu–O bond and points to a more square pyramidal coordination environment rather than an octahedral geometry.^41−44^ Both elemental analysis and single-crystal X-ray crystallography show that a water molecule is coordinated to the copper center, likely originating from the Cu(OTf)_2_ salt, which has a tendency to form hydrates when exposed to air. The coordinated water molecule forms two O–H···O hydrogen bonds (1.980 Å) with one of the oxygen atoms of the axial triflate ligand below the plane and one lattice water solvent molecule. Additionally, the crystal structure confirms that the furanyl group does not coordinate to the Cu center. UV–vis spectra were measured in Milli-Q water, and the extinction coefficient (ε) for the d–d transition at 660 nm is 1.0 × 10^2^ L mol^–1^ cm^–1^, and for the absorption peak at 251 nm, an ε of 9.7 × 10^3^ L mol^–1^ cm^–1^ was found (Supporting Information, Section S2).

### Electrochemistry of Cu-fubmpa, Cu-bpmpa, and Cu-pmea

**Figure 2:**
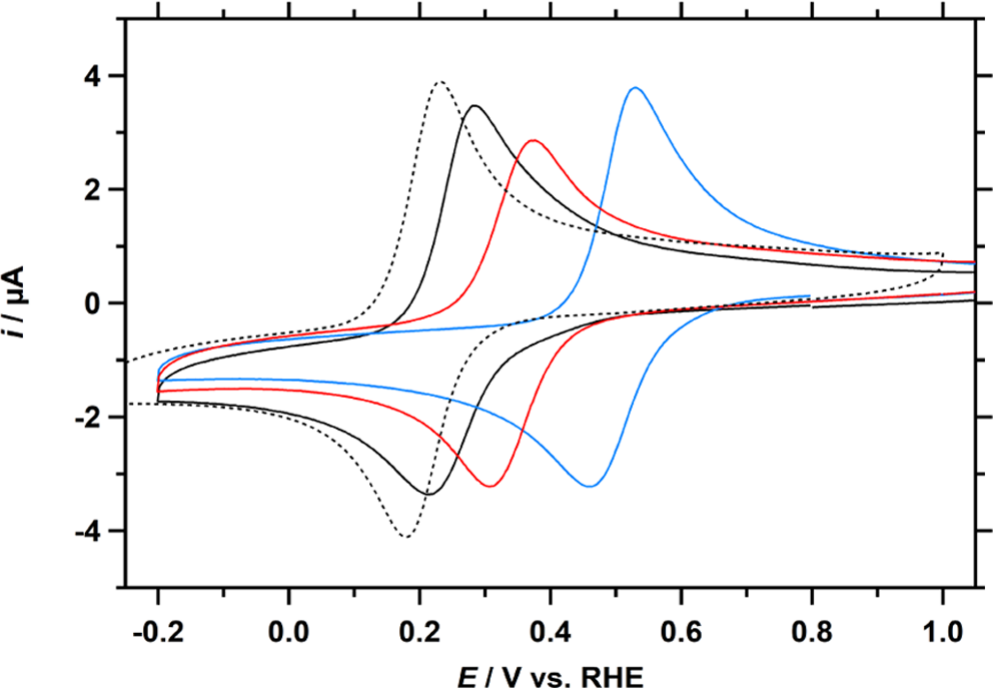
Cyclic voltammograms of Cu-fubmpa (black), Cu-pmea (red), and Cu-bpmpa (blue), including Cu-tmpa (dotted) as a reference, in a pH 7 phosphate buffer under 1 atm Ar. For each copper complex, a concentration of 0.3 mM was used. Conditions: pH 7 PB ([PO_4_] = 100 mM), 293 K, 100 mV s^–1^ scan rate.

To study the effect of the different ligands on the redox chemistry of the complexes, cyclic voltammograms (CVs) of the complexes in a pH 7 phosphate buffer (PB) solution under an argon atmosphere were recorded using a glassy carbon (GC) working electrode (*A* = 0.0707 cm^2^). The resulting redox couples recorded of Cu-fubmpa, Cu-bpmpa, and Cu-pmea with a scan rate of 100 mV s^–1^ are combined in Figure 2, with Cu-tmpa as the reference complex. The *E*_1/2_ of the Cu^II/I^ redox couples of these complexes span a wide potential range (Table 1), shifting positively from the *E*_1/2_ of 0.21 V for Cu-tmpa to 0.25 V for Cu-fubmpa, 0.37 V for Cu-pmea, and 0.49 V for Cu-bpmpa. All three complexes show lower peak currents (*i*_p_) than Cu-tmpa for both the cathodic (*i*_pc_) and anodic (*i*_pa_) peaks, resulting in slightly lower diffusion coefficients (see Table 1 and Supporting Information, Section S4).

**Table 1:** TOF_max_ for the ORR and HPRR Derived from Foot-of-the-Wave Analysis (FOWA)

complex	redox couple		TOF_max_ (s^–1^)	
	*E*_1/2_ (V)	*D* (cm^2^ s^–1^)^b^	ORR	HPRR
Cu-tmpa^a^	0.206	4.9 × 10^–6^	1.8 × 10^6^ ± 0.6 × 10^6^	2.1 × 10^5^ ± 0.1 × 10^5^
Cu-fubmpa	0.248(2)	2.4 × 10^–6^	1.3 × 10^5^ ± 0.3 × 10^5^	0.8 × 10^3^ ± 0.1 × 10^3^
Cu-pmea	0.341(2)	2.9 × 10^–6^	1.4 × 10^3^ ± 0.2 × 10^3^	1.0 × 10^3^ ± 0.3 × 10^3^
Cu-bpmpa	0.494(2)	2.3 × 10^–6^	0.7 ± 0.06	6.4 ± 0.9

a Data from refs (22) and (23).

b Determined from Randles–Sevcik analysis of *i*_pc_. conditions: 0.3 mM catalyst concentration, pH 7 PB ([PO_4_] = 100 mM), 293 K, 100 mV s^–1^ scan rate, 0.0707 cm^2^ electrode surface area.

### Electrocatalytic Performance toward the ORR and HPRR

**Figure 3:**
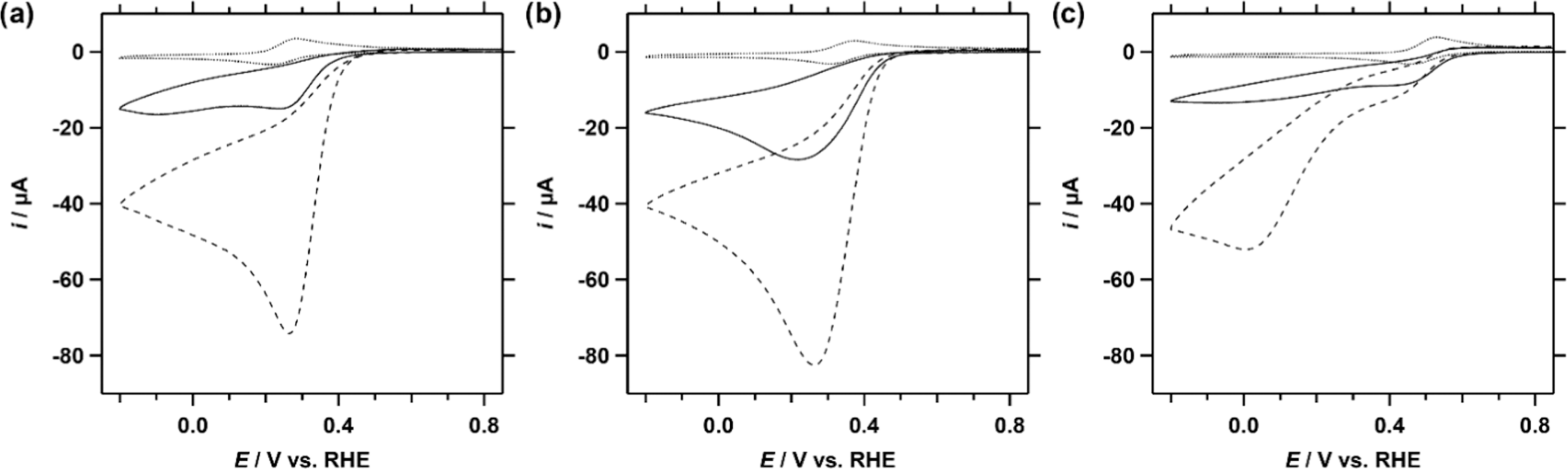
CVs of Cu-fubmpa (a), Cu-pmea (b), and Cu-bpmpa (c) in a PB pH 7 electrolyte solution under 1 atm Ar (dotted line), 1 atm O_2_ (dashed line), or with 1.1 mM H_2_O_2_ under 1 atm Ar (solid line). For each catalyst, a concentration of 0.3 mM was used. Conditions: pH 7 PB ([PO_4_] = 100 mM), 293 K, 100 mV s^–1^ scan rate, 0.0707 cm^2^ electrode surface area.

We have previously shown that Cu-tmpa produces H_2_O_2_ as a detectable intermediate during the electrocatalytic reduction of O_2_, but it can also further reduce H_2_O_2_ to H_2_O.^22,23^ In line with these findings, both the ORR and HPRR were studied for Cu-fubmpa, Cu-bpmpa, and Cu-pmea. CVs were measured in a pH 7 phosphate buffer solution containing 0.3 mM of the complex under 1 atm O_2_ or with 1.1 mM H_2_O_2_ under 1 atm Ar. The resulting catalytic waves for the reduction of O_2_ and H_2_O_2_ are shown in Figure 3 separately for each catalyst. One observation that can immediately be made is that the ORR current is greater than the HPRR current for all of the analyzed complexes. This was also observed for Cu-tmpa previously and most likely related to mass transport limitations in O_2_ and H_2_O_2_. Our experiments are set up such that the local concentrations of O_2_ and H_2_O_2_ are similar (1.2 mM), yet the diffusion coefficient of O_2_ (1.9 × 10^–5^ cm^2^ s^–1^) is significantly larger than that of H_2_O_2_ (0.8–1.4 × 10^–5^ cm^2^ s^–1^).^45,46^ Moreover, the HPRR is a 2-electron process, while the ORR may consume up to 4 electrons, particularly under mass transport-limited conditions. For Cu-fubmpa, the onset of the ORR appears to be ca. 40 mV higher compared with the onset of the HPRR (Figure S19). Here, the onset is defined as *i*_cat_/*i*_p_ ≥ 2 (see the Supporting Information, Table S4). On the other hand, both Cu-bpmpa and Cu-pmea each show overlapping catalytic onsets for the ORR and HPRR. The HPRR onset for Cu-fubmpa is shifted to a lower potential, something that was also observed for Cu-tmpa.^23^

**Figure 4:**
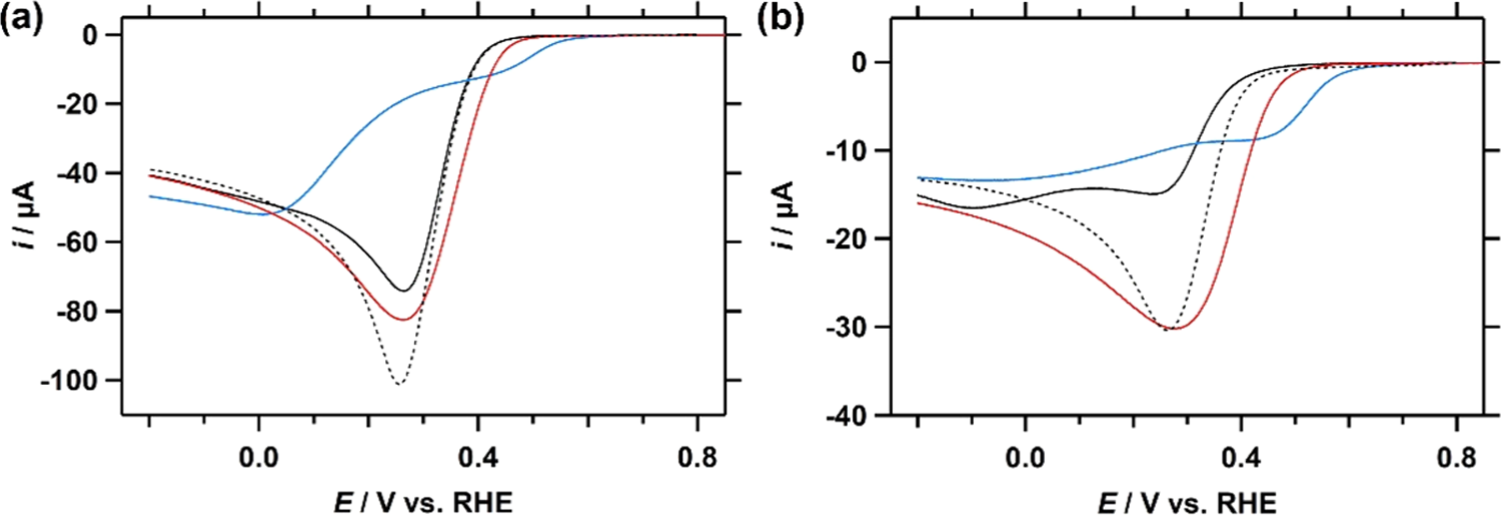
Linear sweep voltammograms (LSV) of Cu-fubmpa (black), Cu-pmea (red), and Cu- bpmpa (blue), including Cu-tmpa (dotted) as a reference, under 1 atm O_2_ (a), or in the presence of 1.1 mM H_2_O_2_ under 1 atm Ar (b). For each catalyst, a concentration of 0.3 mM was used. Conditions: pH 7 PB ([PO_4_] = 100 mM), 293 K, 100 mV s^–1^ scan rate, 0.0707 cm^2^ electrode surface area.

The catalytic linear sweep voltammograms (LSVs) of Cu-fubmpa, Cu-bpmpa, and Cu-pmea complexes of the ORR and HPRR are combined in Figure 4 to allow for a straightforward comparison between the catalysts. The catalytic wave of the ORR in the presence of Cu-fubmpa overlaps neatly with the catalytic wave of Cu-tmpa, while the catalytic onset potential of Cu-pmea is slightly higher. However, both catalysts reach a somewhat lower peak catalytic current (*i*_cat_) than Cu-tmpa. Cu-bpmpa, on the other hand, shows a much earlier onset than the other catalysts, nearer to the 0.695 V vs RHE equilibrium potential of the O_2_/H_2_O_2_ couple. However, a tradeoff for this higher onset potential is the much lower catalytic activity exhibited by the catalyst. In addition, further reduction of the catalytic site appears required before satisfactory reaction rates can be observed (Section S5).

The voltammetry data from the HPRR show a similar trend for the onset potential of the catalytic reaction, with the onset in the presence of Cu-fubmpa < Cu-pmea < Cu-bpmpa (Figure 4b). Of the three catalysts investigated here, fairly similar catalytic currents are observed for Cu-pmea and Cu-tmpa, which is most likely the result of mass transport limitations rather than a true catalytic effect. A lower slope and thus a smaller increase in catalytic rate as a function of applied potential hints at a lower HPRR rate constant for Cu-pmea. The catalytic current of Cu-fubmpa is again significantly lower.

### Correlation between *E*_1/2_ and the Catalytic Rates of ORR and HPRR Using the Foot-of-the-Wave Analysis

The electrochemical reduction of O_2_ proceeds via H_2_O_2_ as an obligatory intermediate in the case of Cu-tmpa^22^ and related pyridylalkylamine complexes.^39,47,48^ Therefore, the electron transfer number is 2 at the foot-of-the-wave, while the electron transfer number is ill-defined at the peak-of-the-wave, where over-reduction to water is likely to occur. We therefore rely for determination of the catalytic rates for the ORR and HPRR on the foot-of-the-wave equation (eq 1), which produces more satisfactory results than the current enhancement method (CE, see the Supporting Information, Section S6). The FOWA extrapolates the ideal or maximum turnover frequency (TOF_max_) of the catalyst from the foot of the catalytic wave, close to the onset of the catalytic reaction (an elaborate detailed description of FOWA was recently given by Dempsey et al.).^49^ An additional advantage of the FOWA method over CE is that it avoids side phenomena, such as O_2_ depletion, which occurs readily due to the limited O_2_ concentration of roughly 1.2 mM at room temperature (293 K) under atmospheric pressure.^50^

For the FOWA, CVs were measured in triplicate in a PB (pH 7) electrolyte solution containing 0.3 mM complex and 1 atm O_2_ (for the ORR), or 1.1 mM H_2_O_2_ in the presence of 1 atm Ar (for the HPRR), using a freshly polished GC electrode for each measurement (see the Supporting Information, Section 6.2). These voltammograms were used to construct plots of the current enhancement *i*_c_/*i*_p_ vs (1 + exp[F/*RT*(*E* – *E*_1/2_)])^−1^, where *i*_c_ is the catalytic current measured in the presence of catalyst and substrate (O_2_ or H_2_O_2_) at the applied potential *E* and *i*_p_ is the peak current of the Cu^II^ reduction in the absence of the substrate. In the foot-of-the-wave potential window, a linear fit was obtained between the catalytic onset and the potential, where *i*_c_/*i*_p_ is at least equal to 1.6. The onset is defined as *i*_c_/*i*_redox_ ≥ 2, where *i*_redox_ is the current measured at the applied potential *E* in the presence of the catalyst but in the absence of the substrate. The TOF_max_ was determined from the slope of the linear fit by applying eq 1. Assumed here is that for fast electrocatalytic reactions, as described here, all electrons necessary to reduce dioxygen (and hydrogen peroxide) come from the electrode and not from neighboring homogeneous sites.^51^ In a previous study,^22,23^ it was already established that in the case of Cu-tmpa, the potential-determining step is reduction of Cu(II) to Cu(I) and that binding of O_2_ to Cu(I) occurs during the rate-determining step in line with a EC′ mechanism.^52^ This signifies that eq 1 is the appropriate FOWA equation in the case of Cu-tmpa. Based on the shape of the FOWA plots (see the Supporting Information), we can assume the same type of reaction mechanism across all catalysts.
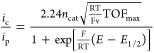
1

**Figure 5:**
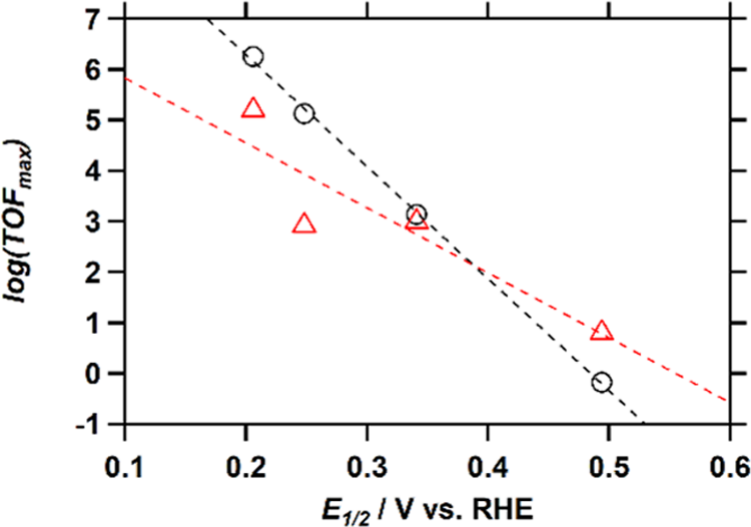
Plot of the logarithm of the TOF_max_ of the ORR (circles; 1 atm O_2_) and HPRR (triangles; 1.1 mM H_2_O_2_) versus the *E*_1/2_ of the respective catalysts, including Cu-tmpa. Linear fit ORR (black dashed line): *y* = – 22.1*x* + 10.7, *R*^2^ = 0.99. Linear fit HPRR (red dashed line): *y* = – 13.1*x* + 7.2, *R*^2^ = 0.82.

The resulting TOF_max_ values for the ORR and HPRR are reported in Table 1. For the ORR, Cu-fubmpa has the highest TOF_max_ (1.3 × 10^5^ s^–1^), while Cu-bpmpa has the lowest (0.7 s^–1^). For the HPRR, Cu-pmea shows the highest TOF_max_, but it is closely followed by Cu-fubmpa. All catalysts discussed here have a TOF_max_ lower than that of the previously reported Cu-tmpa for both catalytic reactions. Comparison of the ORR and HPRR TOF_max_ values reveals an interesting trend. The TOF_max_ of both catalytic reactions decreases with increasing *E*_1/2_ values of the complexes, which results in a change in the relative magnitude of the TOF_max_ of both reactions (Figure 5). For both reactions, a linear fit through the data points was obtained, resulting in an R^2^ value that is close to 1 in the case of the ORR and 0.82 for the HPRR. For Cu-fubmpa, the ORR is much faster than the HPRR, while for Cu-bpmpa, which has the highest *E*_1/2_, the ORR is slower than the HPRR. For Cu-pmea, both reactions show similar TOF_max_ values. Thus, the higher the *E*_1/2_, the more the reduction of H_2_O_2_ seems to be favored over the reduction of O_2_. However, the FOWA does not consider the second, higher catalytic wave observed for Cu-bpmpa in the presence of O_2_, as the TOF_max_ is derived from the initial slope around 0.6 V vs RHE. This second catalytic wave, which is centered at 0.1 V vs RHE, cannot be accurately probed by the FOWA but shows that higher catalytic rates can be achieved in the presence of Cu-bpmpa. This may either point to the formation of a second catalytic species or to reduction of dioxygen via a different reaction mechanism. Either way, the higher catalytic activity observed in the second catalytic wave is at the cost of a significantly increased overpotential. The TOF_max_ values obtained by FOWA, and *k*_obs_ values obtained by CE methods compare well in the case of Cu-tmpa and Cu-fubmpa, yet lead to dissimilar results in the case of Cu-pmea and Cu-bpmpa, which seem to be related to further activation of the catalyst beyond the initial onset of the catalytic wave (see Section S6).

#### O_2_ Binding Constant Determination by DFT

**Figure 6:**
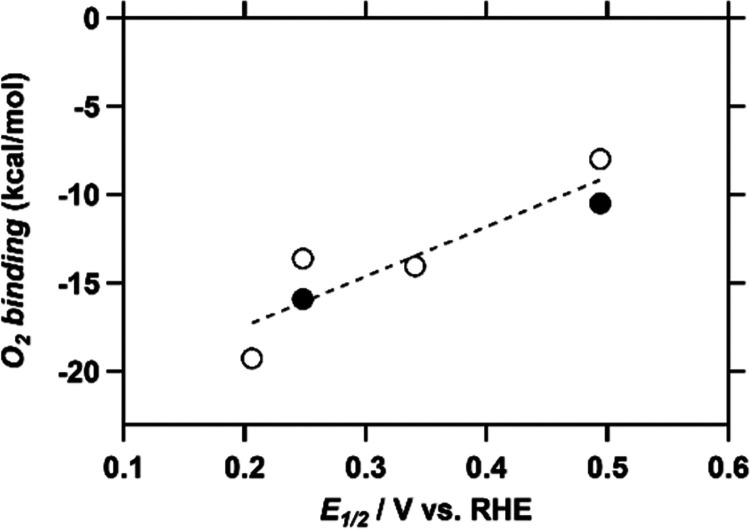
Plot of the calculated binding energies of O_2_ to the Cu(I) state of [Cu(tmpa)]^+^ (*E*_1/2_ = 0.206), [Cu(fubmpa)]^+^ (*E*_1/2_ = 0.248), [Cu(fubmpa)H_2_O]^+^ (*E*_1/2_ = 0.248), [Cu(pmea)]^+^, [Cu(bpmpa)]^+^ (*E*_1/2_ = 0.494), and [Cu(bpmpa)H_2_O]^+^ (*E*_1/2_ = 0.494) versus the *E*_1/2_ of the respective catalysts. For Cu-fubmpa and Cu-bpmpa, the two data points represent the complexes with (solid circle) and without (open circle) a molecule of water coordinated to the copper site. The linear fit was fitted through the data points that represent the complexes without additional water (open circles), with *R*^2^ = 0.84.

Electrochemical methods point to a linear scaling relationship between the log(TOF_max_) values for the ORR and HPRR and the *E*_1/2_ of the catalyst. In order to relate the electronic structure of the catalytic intermediates to the redox potential of the catalyst, density functional theory (DFT) calculations on key catalytic species were carried out (Section S9). In this manner, binding energies of O_2_ to the Cu(I) state of the catalysts could be obtained. Both the triplet state and broken-symmetry singlet state were obtained for the superoxide state of all complexes. In all cases, the triplet state corresponds to the ground state energy of the complexes. This is in line with earlier reports on copper(II) superoxide complexes with side-on O_2_ binding^53^ and previously calculated Cu-tmpa superoxide species.^54^ As depicted in Figure 6, the obtained O_2_ binding energies depend linearly on the *E*_1/2_ since the more electron-rich copper sites tend to bind O_2_ more strongly. In addition, the binding energies of O_2_ to the water adducts of Cu-fubmpa ([Cu(fubmpa)H_2_O]^+^) and Cu-bpmpa ([Cu(bpmpa)H_2_O]^+^) were calculated as their tridentate ligands might provide an extra coordination site for water compared to the tetradentate ligands. These binding energies were excluded from the linear fit but followed the same trend. Previously, it was found that the RDS for the ORR by Cu-tmpa is most probably the binding of O_2_.^22^ Based on the trends displayed in Figure 5, this hypothesis can be further extended to Cu-fubmpa, Cu-pmea, and Cu-bpmpa, as both O_2_ binding and TOF_max_ now scale with *E*_1/2_. Therefore, this finding provides a rationale for the reaction rate, which thus depends on the binding energy of O_2_, which is determined by the *E*_1/2_.

Regarding the binding of H_2_O_2_ to the Cu(I) states of all catalysts, the differences in binding energies are small and fall within the error range of the computations (see Section S9). In line with our previously published results, H_2_O_2_ binding probably occurs in a pre-equilibrium, while the rate-determining step of HPRR is probably associated with O–O bond scission.^9−23^

#### Rotating Ring-Disk Electrode Measurements

Interestingly, Figure 5 shows that at a certain *E*_1/2_ value, the relative activities for the ORR and HPRR invert, resulting in the HPRR becoming the faster catalytic reaction of the two as the *E*_1/2_ of the catalyst increases. This implies that the *E*_1/2_ of the Cu complex must be a key descriptor regarding the selectivity of the ORR and generation of H_2_O_2_ mediated at single-site copper species. To investigate this hypothesis, rotating ring-disk electrode (RRDE) measurements were performed. These measurements allow us to determine the selectivity of all catalysts for the overall 2- vs 4-electron reduction of oxygen, as the hydrogen peroxide that is generated at the disk can be detected and quantified at a Pt ring. RRDE measurements of Cu-pmea, Cu-bpmpa, and Cu-fubmpa were recorded and compared to the previously reported RRDE data of Cu-tmpa.^22,23^ LSV measurements in 0.1 M PB show for Cu-fubmpa and Cu-pmea a catalytic current that reaches a limiting plateau current below 0.2 V vs RHE. In the case of Cu-bpmpa, a plateau current is not reached; instead, the LSV shows a small catalytic wave, followed by a second larger catalytic wave at a much lower potential (Figure S21), which is in line with the CV data from Figure 3c. Analysis of the catalytic currents at different rotation rates shows for all catalysts that the current linearly depends on the square root of the rotation, indicating that the number of electrons transferred does not depend on rotation speed (see Figure S23). In addition, the onset potential of the catalytic ORR was determined in the same manner for stationary CV experiments and followed the same trend (Figure S24 and Table S4).

**Figure 7:**
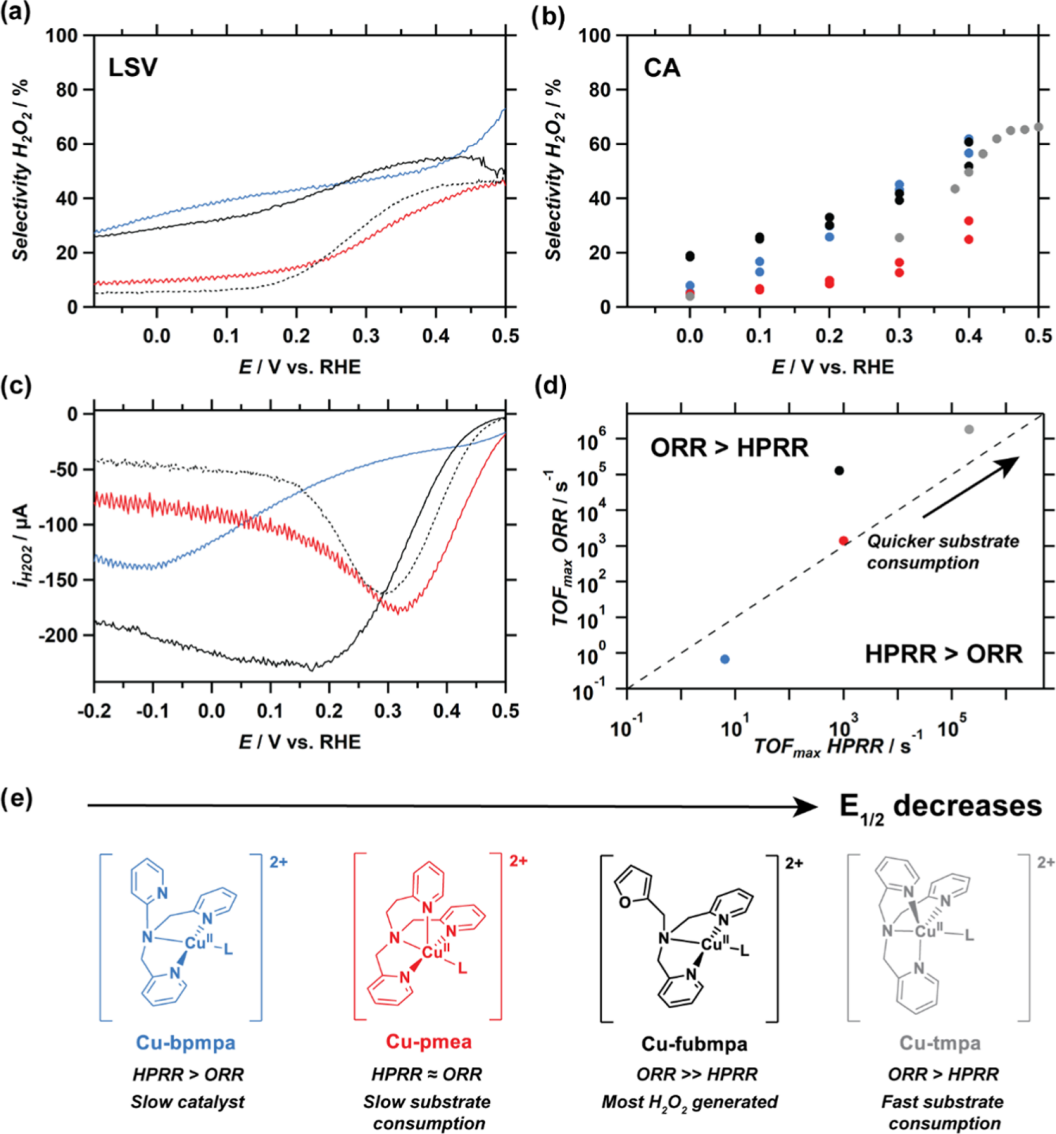
Selectivity of the ORR determined from RRDE LSV (a) and CA (b) experiments and catalytic current to H_2_O_2_ (2-electron ORR) determined from RRDE LSV data (c) catalyzed by Cu-fubmpa (black), Cu-bpmpa (blue), Cu-pmea (red), and Cu(tmpa) (gray dots/dotted line) under 1 atm O_2_. (d) Compared to a plot of the TOF_max_ values determined for ORR and HPRR for Cu-fubmpa (black), Cu-bpmpa (blue), Cu-pmea (red), and Cu(tmpa) (gray) as shown in Table 1. Data for Cu-tmpa obtained from ref (22). Catalyst structures and the most important conclusions are shown in panel (e). A catalyst concentration of 0.3 mM was used for each complex. Conditions: pH 7 PB ([PO_4_] = 100 mM), 293 K, 0.196 cm^2^ electrode surface area, 1600 RPM, Pt ring at 1.2 V vs RHE.

Subsequently, analysis of the current measured at the Pt ring allows us to determine the selectivity of the catalysts during catalysis of the ORR and verifies that H_2_O_2_ is produced as an (intermediate) product (see Figure S25). Figure 7a,b shows the selectivity of the ORR determined from the LSV and chronoamperometry (CA) experiments for all four catalysts. In general, the LSV and CA data follow the same trends, but there is a deviation between the exact values, caused by the different nature of both experiments. To make sure that the H_2_O_2_ selectivity is determined correctly, the reproducibility of both measurements was verified (see Figure S21). Besides the selectivity, the catalytic current that is used to convert oxygen to hydrogen peroxide (*i*_H_2_O_2__) can be determined from RRDE measurements as well, as shown in Figure 7c. The trends on H_2_O_2_ selectivity and *i*_H_2_O_2__ for the different catalysts can be explained with the help of Figure 7d, in which the TOF_max_ of the ORR is plotted vs the TOF_max_ of the HPRR, as determined by FOWA (see Table 1). This graph indicates to what extent one of these two reactions proceeds faster. In general, for the ORR > HPRR regime, the TOF_max_ of the ORR is higher than that of the HPRR and H_2_O_2_ is generated faster than it can be consumed, while for the HPRR > ORR regime, the opposite is true. On top of this, it is important to note that for faster catalysts, the consumption of the substrate, both O_2_ and H_2_O_2_, will be quicker. As a consequence, faster catalysts will quickly consume O_2_, which will result in over-reduction of H_2_O_2_ to H_2_O, at the cost of a lower H_2_O_2_ selectivity. We can now rationalize the trends in selectivity and *i*_H_2_O_2__ in Figure 7 by considering the relative rates of the ORR and HPRR in Figure 7d.

Starting from the catalyst with the highest *E*_1/2_, Cu-bpmpa is not much more active than the bare GC electrode, especially at the start of the catalytic wave. The small quantities of H_2_O_2_ detected at the ring may in part originate from the bare GC electrode, which makes the interpretation of the data difficult. Only at potentials below 0.0 V vs RHE, the catalyst is clearly more active than the GC electrode and the H_2_O_2_ selectivity of Cu-bpmpa instantly starts to drop (see Figure S22). The small *i*_H_2_O_2__ and low H_2_O_2_ selectivity are expected, as this catalyst is slowest for both ORR and HPRR, with the HPRR rate being higher than the ORR rate. Next, Cu-pmea is a more active catalyst for both the ORR and HPRR compared to Cu-bpmpa, resulting in a larger *i*_H_2_O_2__. This catalyst produces H_2_O_2_ close to the onset of the ORR, but both selectivity to H_2_O_2_ and *i*_H_2_O_2__ drop at lower potentials. This can be explained by the HPRR rate being close to the ORR rate. The TOF_max_ of the HPRR for Cu-pmea is comparable to that of Cu-fubmpa. However, for Cu-fubmpa, the rate of the ORR is much higher than that of the HPRR. This results in a high selectivity to H_2_O_2_ and a large *i*_H_2_O_2__ over the whole potential window compared to the other catalysts. Lastly, for Cu-tmpa, the ORR rate is higher than the HPRR at the same substrate concentrations. In addition, the rates for both ORR and HPRR are much faster compared to the other catalysts. As a result, Cu-tmpa will quickly convert most O_2_ to H_2_O_2_, while, in turn, this H_2_O_2_ is also quickly consumed to generate H_2_O, as shown previously.^22,23^ This results in a H_2_O_2_ selectivity that is, in general, lower than that for Cu-fubmpa. Taken together, we can explain the RRDE results in terms of product selectivity and *i*_H_2_O_2__ based on the TOF_max_ of the ORR and HPRR, which are in turn linked to the *E*_1/2_ of these catalysts. From the RRDE data, the most H_2_O_2_ is generated in the case of Cu-fubmpa, which has a relatively high ORR rate compared to the HPRR. In turn, 4-electron reduction of oxygen can either be achieved by a catalyst that has a similar TOF_max_ for the HPRR and ORR, as is the case for Cu-pmea, or by a catalyst that will quickly consume all O_2_, ultimately leading to further reduction of H_2_O_2_ to H_2_O, which is the case for Cu-tmpa.

## Discussion

Variation in the length of the (–CH_2_)*_n_* spacer (where *n* = 0–2) between the central tertiary amine and one of the pyridine moieties results in a significant shift in the equilibrium potential of the Cu^II^/Cu^I^ redox couple. These observations are fully in line with the results of the Rorabacher and Karlin groups in the past, who have shown a clear correlation between the ligand-ring size and the Cu^II^/Cu^I^ redox couple.^26,27,55,56^ The shifts of Cu-pmea and Cu-bpmpa toward a higher potential are much larger than observed for Cu-fubmpa, in which one of the pyridine arms is replaced for a furanyl group, thereby keeping the central tertiary amine intact while preventing the coordination of a third ligand arm to the Cu center. In this way, the effect of a lower denticity on the catalytic activity could be investigated without removing the pyridine arm entirely, as this would have introduced a secondary amine that could be easily oxidized during the catalytic cycle. Indeed, the *E*_1/2_ of Cu-fubmpa and Cu-bmpa (bmpa = bis(2-pyridylmethyl)amine) is similar in a pH 7 phosphate buffer (the *E*_1/2_ of Cu-bmpa is 0.30 V vs RHE),^39^ indicating that the coordination of the furanyl group does not occur while in solution.

A linear relationship between the maximum TOF [log(TOF_max_)] and the *E*_1/2_ of the catalytic species is observed, as visualized in Figure 5. As the catalyst *E*_1/2_ increases and thus the overpotential decreases, the rate of the reaction decreases. This behavior seems to hold for both the ORR and the HPRR, although the effect is smaller with more deviations in the case of the HPRR TOF_max_. Such Evans–Polanyi type scaling relations between the log rate and typically the overpotential have been reported previously in the case of some very well-behaved electrocatalysts.^57−61^ Typically, these scaling relations are plotted versus the overpotential of the catalytic reaction.^47,58−60^ We believe that in this context, the *E*_1/2_ is a more appropriate descriptor than, for example, the overpotential that is frequently used given that the overpotentials of ORR and HPRR are often ill-defined if these are not fully under a kinetic control. Moreover, the relationship versus *E*_1/2_ allows for a more facile comparison between the ORR and HPRR results, which have different standard reduction potentials. Not only log TOF_max_ but also the computed binding constant of O_2_ to the various copper site correlates well with the *E*_1/2_ value of these copper sites. These correlations are in perfect agreement with a potential-determining reduction of Cu^II^ to Cu^I^, followed by rate-limiting binding of O_2_ for the entire series of copper species investigated here. In the case of Cu-tmpa, these potential- and rate-determining steps were already identified on basis of kinetic studies discussed in previous reports.^22,23,62^ It is important to note that also the data previously obtained for Cu-bmpa (*E*_1/2_ = 0.30 V vs RHE, TOF_max_ for ORR = 2.4 × 10^4^ s^–1^) correlates very well with the ORR TOF_max_ versus *E*_1/2_ trend reported here,^39^ while catalysts that show very sluggish and therefore rate-determining Cu(II) reduction kinetics do significantly underperform as one would expect.^39,47,63,64^

Displacement of water for peroxide presumably takes place prior to the rate-determining step of the HPRR, and with similar energetics for all copper complexes, unlike the binding of dioxygen. The relative insensitivity of H_2_O_2_ versus H_2_O binding to these copper sites is to be expected, as both species bind to the copper site in a very similar manner. The actual rate-determining step most likely involves the scission of the O–O bond, presumably via a Fenton-like reaction.^23^ This reaction is highly exothermic and occurs far from the equilibrium potential of peroxide (*E*_H_2_O_2_/H_2_O_^0^ = 1.78 V vs RHE) and therefore is expected to be less dependent on the electronic structure of the copper site.

Interestingly, the HPRR and ORR scale differently with *E*_1/2_ of the catalyst. This implies that the *E*_1/2_ of the Cu complex must also be a key descriptor regarding the selectivity of the ORR mediated at single site copper species. Our findings from RRDE measurements illustrate that electron-rich copper sites are set up to produce significant amounts of peroxide with fast reaction rates, while electron-poor copper sites will react slower in the ORR and preferably reduce hydrogen peroxide over dioxygen, thus favoring the ultimate formation of water. In practice, it is not always easy to visualize in a single experiment that the ORR selectivity directly correlates to *E*_1/2_, given that the overall rates of the catalysts are largely different, thereby resulting that these catalysts arrive at a mass transport-limited regime at a different moment. When this is taken into account, a clear correlation between *E*_1/2_, the ORR and HPRR rates, and the selectivity can be drawn. Catalysts that operate at higher potentials due to a more positive *E*_1/2_ are expected to reduce dioxygen in an overall 4-electron reduction reaction given that their HPRR rates are significantly larger than the ORR rates, but, in practice, catalytic rates mediated by these species will be sluggish. Consequently, finding a catalyst that can produce hydrogen peroxide selectively near the equilibrium potential of the peroxide will be hard. Catalysts that operate at more negative potentials show a very high affinity for O_2_ binding and show very high catalytic rates toward the formation of hydrogen peroxide, yet such catalysts are easily limited by mass transport limitations. When mass transport limitations occur, and locally, most O_2_ is consumed, all catalysts are expected to preferentially catalyze the full 4-electron reduction to water.

## Conclusions

We have investigated the correlation between *E*_1/2_ and the catalytic performance in the oxygen and hydrogen peroxide reduction reactions for a series of copper complexes based on the tetradentate tmpa-based ligand scaffold. Our findings show that the log(TOF_max_) of the ORR and HPRR correlates linearly with *E*_1/2_. A direct correlation between the computed O_2_ binding constant to *E*_1/2_ was found as well and is in good agreement with rate-determining binding of O_2_ to a Cu^I^ species. Since the ORR and HPRR scale versus *E*_1/2_ with significantly different slopes, the *E*_1/2_ value is a leading descriptor for the selectivity of the oxygen reduction process. Our design rules are the following: Copper species with more positive *E*_1/2_ values are expected to catalyze the overall 4-electron reduction reaction to water more selectively, yet achieving high catalytic rates for such a catalyst will be challenging; catalysts with more negative *E*_1/2_ values are likely to accumulate hydrogen peroxide, providing that mass transport limitations can be avoided.

## Supporting Information Available

Experimental procedures, spectra and crystallographic data, additional voltammetry data and details on determination of the catalytic rate by foot-of-the-wave, current enhancement methods, rotating ring-disk experiments, and computational details (PDF)
