# Supplementary material for: Scaling Relation between the Reduction Potential of Copper Catalysts and the Turnover Frequency for the Oxygen and Hydrogen Peroxide Reduction Reactions
Source: Inorg Chem. 2023 Nov 17;62(48):19593–602. doi: 10.1021/acs.inorgchem.3c02939 (PMC10698719; doi:10.1021/acs.inorgchem.3c02939)
Supplement: Supplementary file 1 — ic3c02939_si_001.pdf [file ic3c02939_si_001.pdf]

Supporting Information

On the scaling relation between the reduction potential of copper catalysts and the turnover frequency for the oxygen and hydrogen peroxide reduction reactions

*Michiel Langerman,[a] Phebe H. van Langevelde,[a] Johannes J. van de Vijver,[a] Maxime A. Siegler,[b] and Dennis G. H. Hetterscheid[a]**

[a] Leiden Institute of Chemistry, Leiden University, Einsteinweg 55, 2300 RA, Leiden, The Netherlands

[b] Department of Chemistry, Johns Hopkins University, 3400 North Charles St., Baltimore, Maryland 21218, United States

**Corresponding author:* d.g.h.hetterscheid@chem.leidenuniv.nl

Contents

S1. Experimental Section – Page S3

S2. Spectra belonging to fubmpa and Cu-fubmpa – Page S9

S3. Crystallography data of Cu-fubmpa – Page S15

S4 Data obtained from Cyclic Voltammetry – Page S18

S5. Catalytic performance of Cu-bpmpa – Page S21

S6. Determination of the catalytic rate – Page S23

S6.1 Current enhancement method

S6.2 Foot-of-the-wave analysis

S7. Rotating Ring Disk Electrochemistry measurements – Page S37

S8. H_2O_2 selectivity derived from RRDE measurements – Page S43

S9. Computational methods – Page S46

S10. References – Page S51

S1. Experimental Section

General Procedures. All precursors used in the ligand synthesis were obtained from Sigma-Aldrich/Merck. $Cu(OTf)_2$ was obtained from Alfa Aesar. All other chemicals and solvents were purchased from commercial suppliers. Whatman® RC60 membrane filters were used for the filtration and isolation of the synthesized copper complexes. NMR spectra were recorded on a Bruker 400 MHz or Bruker DPX 300 spectrometer. ESI MS mass spectra were obtained on a Thermo Fisher Scientific MSQ Plus. UV-vis spectra were recorded on a Varian Cary® 40 UV-vis spectrophotometer, or a HORIBA Aqualog spectrophotometer. Elemental analysis was performed by Mikroanalytisches Laboratorium Kolbe. Aqueous electrolyte solutions were prepared using NaH_2PO_4 (Suprapur®, Merck) and Na_2HPO_4 (Suprapur®, Merck). Milli-Q Ultrapure grade water was used in all electrochemical experiments and for the preparation of all aqueous electrolyte solutions. H_2O_2 was obtained from Sigma-Aldrich (≥30%, for ultratrace analysis), and the exact concentration was determined via permanganate titration. pH measurements were performed on a Hanna Instruments HI 4222 pH meter which was calibrated by five-point calibration using IUPAC standard buffers. Alumina suspensions (1.0, 0.3, and 0.05 μm) for electrode polishing were obtained from Buehler. All gasses used during electrochemical measurements, H_2, O_2, and argon (each 5.0 grade), were supplied by Linde.

Synthesis of N-(Furan-2-ylmethyl)-N-[bis(2-pyridyl)methyl]amine (fubmpa). 2-pyridine carboxaldehyde (0.38 mL, 4 mmol) and furan-2-ylmethanamine (0.18 mL, 2 mmol) were added to dry 1,2-dichloroethane (10 mL) and stirred for 1 hour. Sodium triacetoxyborohydride (1.27 g, 6 mmol) was added and the mixture was stirred for 24 hours at room temperature under N_2 atmosphere. $NaHCO_3$ (sat.aq.; 10 mL) was added to the mixture and stirred for one hour to quench the reaction. The organic layer was then separated, concentrated and the residue was purified over alumina column eluting with EtOAc/PetEt (40-60)/MeOH = 50:50:0.5. After removal of the solvent by rotary evaporation at reduced pressure, the product was obtained as a pale, yellow oil (0.39 g, 1.4

mmol, 69% yield). ESI MS m/z (found (calc)): 280.0 (280.1, $[M + H^+]^+$). ^{1}H NMR (400 MHz, $CDCl_3$) δ 8.52 (ddd, *J* = 4.9, 1.8, 1.0 Hz, 2H, py-*H*6), 7.65 (ddd, *J* = 7.8, 7.4, 1.8 Hz, 2H, py-*H*4), 7.58 (ddd, *J* = 7.8, 1.2, 1.0 Hz, 2H, py-*H*3), 7.38 (d, *J* = 1.8 Hz, 1H, fu-*H*5), 7.13 (ddd, *J* = 7.4, 4.9, 1.2 Hz, 2H, py-*H*5), 6.30 (dd, *J* = 3.2, 1.8 Hz, 1H, fu-*H*4), 6.23 (d, *J* = 3.2 Hz, 1H, fu-*H*3), 3.85 (s, 4H, py-CH_2), 3.74 (s, 2H, fu-CH_2). ^{13}C NMR (101 MHz, $CDCl_3$) δ 159.5 (py-*C*2), 152.1 (fu-*C*2), 149.0 (py-*C*6), 142.1 (fu-*C*5), 136.5 (py-*C*4), 122.9 (py-*C*3), 122.0 (py-*C*5), 110.1 (fu-*C*4), 109.0 (fu-*C*3), 59.7 (py-CH_2), 50.4 (fu-CH_2).

Synthesis of N-[bis(2-pyridyl)methyl]-2-pyridylamine (bpmpa). A modified literature procedure was used for the synthesis of bpmpa.[1] Sodium hydride (60% in mineral oil; 0.72 g, 18 mmol) was added to anhydrous DMF (15 mL) under N_2. 2-aminopyridine (0.29 g, 3 mmol) was dissolved in anhydrous DMF (15 mL) under N_2 and subsequently added to the sodium hydride solution and stirred for 30 minutes. 2-chloromethylpyridine•HCl (984 mg, 6 mmol) in anhydrous DMF (20 mL) was added dropwise to the solution over a period of 30 minutes under N_2. The solution was heated to 50 °C and stirred for 24 hours. The resulting black solution was carefully quenched with water (50 mL) which resulted in a yellow solution. A saturated $NaHCO_3$ solution (50 mL) was added, followed by extraction with DCM (4 × 120 mL). The combined organic fractions were subsequently washed with more saturated $NaHCO_3$ solution (3 × 50 mL). The solvent was evaporated by rotary evaporation under reduced pressure and the resulting dried crude product was further purified by silica column chromatography. The crude was dissolved in a few milliliters of DCM, loaded on silica, and the product was obtained with a 98:2 DCM/MeOH mixture as eluent. The product fractions were combined, and the solvent removed by rotary evaporation under reduced pressure. The product was obtained as a slightly yellow oil (0.44 g, 1.6 mmol, 53% yield). ^{1}H NMR (400 MHz, $CDCl_3$) δ 8.53 (dd, *J* = 5.0, 1.9, 2H), 8.17 (ddd, *J* = 5.0, 2.0, 1.0 Hz, 1H), 7.58 (td, *J* = 7.7, 1.9 Hz, 2H), 7.37 (ddd, *J* = 8.6, 7.2, 1.9 Hz, 1H), 7.22 (d, *J* = 7.7 Hz, 2H), 7.13 (dd, *J* = 7.7, 5.0, 2H), 6.59 (7.2, 5.0, 1H), 6.47 (dd, *J* = 8.6, 1.0 Hz, 1H), 4.96 (s, 4H).

Synthesis of bis[(2-pyridyl)methyl]-2-(2-pyridyl)ethylamine (pmea).[2] Pyridine-2-carboxyaldehyde (2.25 g, 21 mmol, 2 equiv.) was added to a stirred mixture of 2-(2-pyridyl)ethylamine (1.28 g, 10.5 mmol, 1 equiv.) and sodium triacetoxyborohydride (6.21 g, 21 mmol, 1 equiv.) in dry EDC (100 mL). Molecular sieves were added to remove H_2O during the reaction. This mixture was stirred under argon for seven days. Subsequently, $NaHCO_3$ (sat.aq.; 100 mL) was added and the mixture was stirred for 30 minutes. The mixture was filtered to remove molecular sieves. The crude mixture was then washed with $NaHCO_3$ (sat.aq) (2x 50 mL) and the organic phase was dried with $MgSO_4$ and filtered again. The EDC was evaporated at 40 °C by rotary evaporation. The crude was dissolved in DCM and extracted with an aqueous solution of pH 4 (acidified with HCl; 3 × 30 mL). The pH of the combined aqueous solution was increased to pH 9 by addition of saturated $NaHCO_3$ and extracted with DCM (6 × 50 mL). The combined organic layers were dried with Na_2SO_4 followed by filtration. The solvent was removed by rotary evaporation at reduced pressure. TLC (Aluminium oxide; 100:10 EtOAc/MeOH) revealed the presence of some impurities close to the baseline. The crude was dissolved in a few millilitres of DCM, loaded on Aluminium oxide, and the product (R_f = 0.7) was obtained using a 100:10 EtOAc/MeOH mixture as eluent. The product fractions were combined, and the solvent removed by rotary evaporation under reduced pressure. The resulting brown oil was exhaustively extracted with warm pentane. Evaporation of the pentane resulted in a colourless oil (1.9 g, 6.3 mmol, 60% yield). ESI MS m/z (found (calc)): 305.2 (305.2, $[M + H^+]^+$), 327.1 (327.2, $[M + Na^+]^+$). ^1H NMR (300 MHz, $CDCl_3$) δ 8.53 – 8.42 (m, 3H), 7.54 (qd, J = 7.5, 1.8 Hz, 3H), 7.33 (dt, *J* = 7.8, 1.1 Hz, 2H), 7.09 (m, 4H), 3.87 (s, 4H, CH_2), 3.08 – 2.89 (m, 4H, CH_2-CH_2).

Synthesis of [Cu(fubmpa)(H₂O)(OTf)₂]. Fubmpa (0.2 g, 0.716 mmol) and $Cu(OTf)_2$ (0.259 g, 0.716 mmol) were dissolved in CH_3CN (10 mL) and together stirred for 1 hour. Following this, the solvent was removed by rotary evaporation at reduced pressure and the complex was dissolved in a minimal amount of CH_3CN until fully dissolved. Diethyl ether was slowly added until the solution became

clouded, upon which a few drops of CH_3CN were added to make sure the complex was fully dissolved, and the solution remain homogenous. The solution was put in the freezer at −18 °C for the crystals to form. This crystallization was done twice to make sure the complex was pure. After filtration of mixture, the complex; [**1**]$(OTf)_2$ was obtained as a navy blue solid (0.32 g, 0.48 mmol, 67%). ESI MS m/z (found (calc)): 211.9 (212.0 $[M - OH_2 - 2OTf + 2MeCN]^{2+}$), 387.0 (387.1 $[M - OH_2 - 2OTf + HCOO^{-}]^{+}$). Elemental analysis calcd (%) for $C_{19}H_{19}CuF_6N_3O_8S_2$: C 34.63, H 2.91, N 6.38; found: C 34.27, H 3.04, N 6.66. UV-Vis: λ_{max} (ε in L mol^{-1} cm^{-1}) in milliQ water = 251 nm (9740); 660 nm (99).

Synthesis of [Cu(bpmpa)(CH_3CN)]($OTf)_2$. $Cu(OTf)_2$ (1.5 mmol, 542 mg) was dissolved in dry CH_3CN (5 mL) under N_2 atmosphere. and bpmpa (1.5 mmol, 0.42 g) in dry CH_3CN (30 mL) was subsequently added to the solution. A dark green solution formed immediately, and the solution was stirred for 1 hour. The solvent was removed by rotary evaporation under reduced pressure and the dark green solid was dissolved in a minimal amount of CH_3CN until fully dissolved. Diethyl ether was added until the solution became clouded, after which a few drops of CH_3CN were added to make sure the complex was fully dissolved. The solution was put in the freezer at −18 °C for 3 days, allowing for crystallization of the complex. The dark turquoise crystals were filtered off and washed with Et_2O. Yield: 72% (1.1 mmol, 0.73 g). ESI MS m/z (found (calc)): 210.6 (210.5 $[M - 2OTf + 2MeCN]^{2+}$), 384.0 (384.1 $[M - 2OTf + HCOO^{-}]^{+}$). Elemental analysis calcd (%) for $C_{21}H_{19}CuF_6N_5O_6S_2 + 0.5\ H_2O$: C 36.66, H 2.93, N 10.18, found values: C 36.50, H 2.83, N 10.08.

Synthesis of [Cu(pmea)(CH_3CN)]($OTf)_2$. $Cu(OTf)_2$ (3 mmol, 913 mg) was dissolved in dry CH_3CN (5 mL) under N_2 atmosphere. and bpmpa (3 mmol, 1.09 g) in dry CH_3CN (30 mL) was subsequently added to the solution. A dark turquoise solution formed immediately, and the solution was stirred for 1 hour. The solvent was removed by rotary evaporation under reduced pressure. The crude complex was dissolved in minimal amounts of $CHCl_3$ until fully dissolved. Methyl tert-butyl ether (MTBE) was added until the solution became clouded, after which a few drops of $CHCl_3$ were added to make

sure the complex was fully dissolved. The solution was put in the freezer at −18 °C for 7 days, allowing for crystallization of the complex. The solution separated into a dark blue crystalline solid and a small amount of a green oil-like substance. The supernatant, including the green oil, were decanted from the round-bottom flask, and the remaining blue crystalline solid was washed with a 30:70 $CHCl_3$/MTBE (50 mL) and filtered off. The solid was crushed into smaller pieces and dried under vacuum. Yield: 82% (2.5 mmol, 1.74 g). ESI MS m/z (found (calc)): 204.2 (204.0 $[M - 2OTf]^{2+}$), 412.1 (412.1 $[M - MeCN- 2OTf + HCOO^-]^+$), 516.0 (516.1 $[M – MeCN - OTf]^+$). Elemental analysis calcd (%) for $C_{23}H_{23}CuF_6N_5O_6S_2 + H_2O$: C 38.10, H 3.48, N 9.66; found: C 37.78, H 3.22, N 9.41.

Electrochemical measurements. All stationary electrochemical experiments were performed using a custom-built 10 mL single-compartment glass cell with a three-electrode setup. The measurements were performed using Autolab PGSTAT 12, 204, and 128N potentiostats, operated by the Autolab NOVA 2 software. The working electrode is a PEEK encapsulated GC disk (A = 0.071 cm^2, Metrohm) submerged in the solution. Unless otherwise stated, the GC electrode was manually polished before each catalytic measurement for 5 mins with 1.0, 0.3, and 0.05 μm alumina suspensions on Buehler cloth polishing pads, or with a Struers LaboPol-30 polishing machine using 1.0 μm diamond and 0.04 μm silica suspension on polishing cloths (Dur-type) for 1 min each. This was followed by sonication of the electrode in Milli-Q purified water for 10–15 minutes. A gold wire was used as a counter electrode and was flame annealed and rinsed with Milli-Q purified water. The reference electrode was a reversible hydrogen electrode (RHE) made from a Pt mesh submerged in same electrolyte solution as the main cell compartment, connected via a Luggin capillary, and the reference compartment was continuously saturated with H_2 gas. Oxygen-free electrolyte solutions were prepared by saturating the cell for 20 to 30 minutes with Ar, after which an atmosphere of 1 atm Ar was maintained over the solution. Oxygen-saturated electrolyte solutions were obtained by

saturating the cell for 20 minutes with O_2, after which a 1 atm O_2 atmosphere was maintained over the solution.

Prior to each experiment, the glassware was fully submerged and boiled in MilliQ purified water. Additionally, all glassware was regularly cleaned by submersion in an aqueous oxidizing solution containing 0.5 M H_2SO_4 and 1 mg/mL (6.3 mM) $KMnO_4$ overnight. This is followed by removal of excess $KMnO_4$ and MnO_2 from the glassware with diluted H_2SO_4 and H_2O_2, followed by rinsing the glassware three times with water and boiling twice submerged in Milli-Q purified water.

For RRDE experiments, a procedure similar to stationary electrochemical measurements was followed. A MSR rotator from Pine Instruments was used in combination with an Autolab PGSTAT 12 potentiostat. The Au counter electrode and RHE reference electrode were prepared as described above. As working electrode, a ChangeDisk RRDE electrode (E6R1PK), with a PEEK shroud, GC disk ($A = 0.196$ cm^2) and Pt ring, was used. The GC disk and Pt ring were polished separately on a Struers LaboPol-30 polishing machine with diamond polish (1.0 μm, 3 minutes) and silica suspension (0.04 μm, 3 minutes) on polishing cloths (Dur-type) and sonicated in Mili-Q water for 15 minutes. All glassware was cleaned as described above, but a 40 mL custom-built electrochemical cell was used. Prior to all RRDE experiments the electrolyte solution was bubbled with O_2 gas for at least 25 minutes, and during the experiment O_2 gas was also bubbled through the solution.

S2. Spectra belonging to fubmpa and Cu-fubmpa

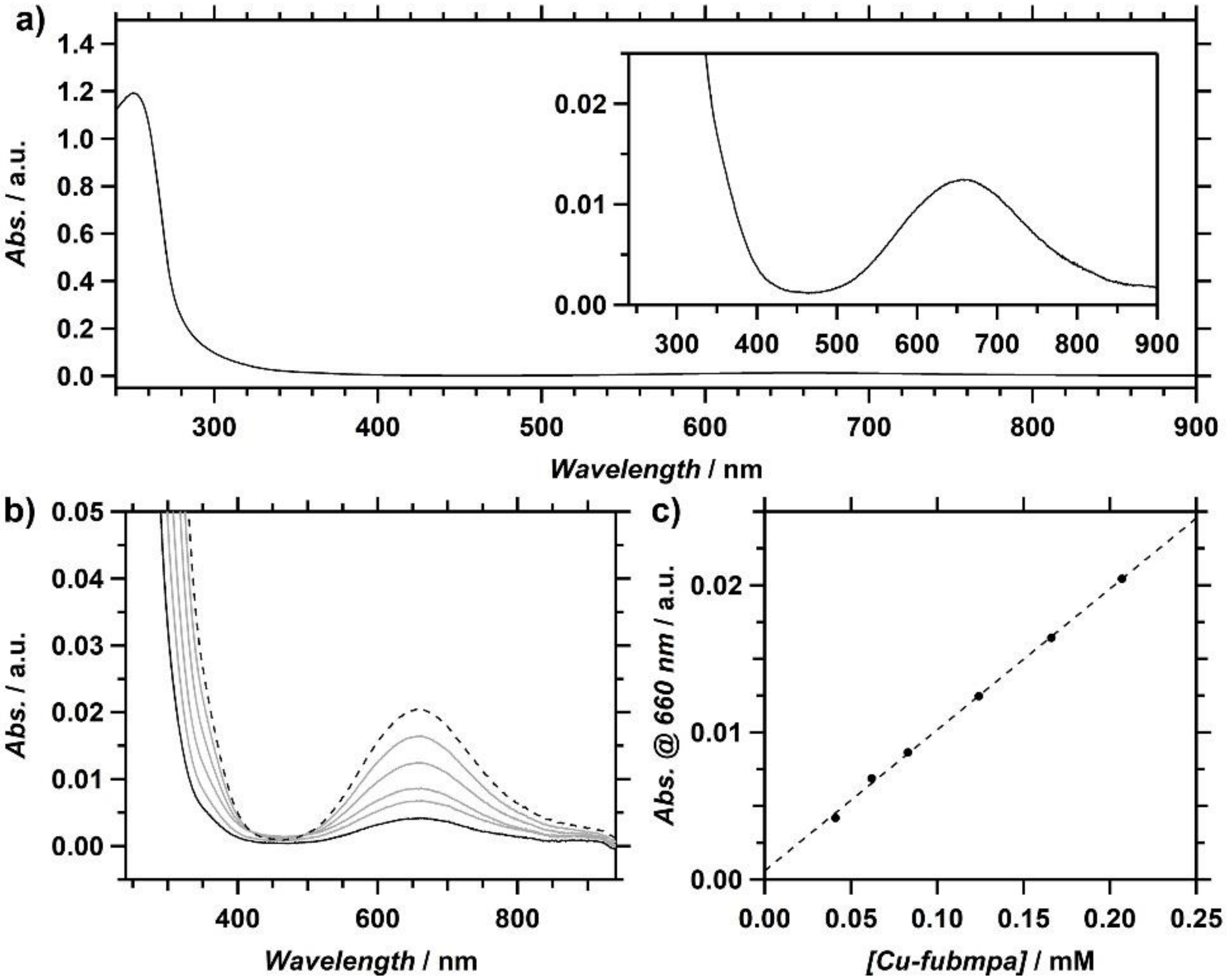

Figure S1. a) UV-vis spectrum of 0.12 mM Cu-fubmpa in water (MilliQ). The inset shows the Cu^{II} d-d transition. **b)** UV-vis spectra of 0.04 (solid black) to 0.2 (dashed) mM Cu-fubmpa in water (MilliQ). **c)** The linear relationship between catalyst concentration and the peak absorbance at 660 nm. Conditions: 293 K, 10 mm path length.

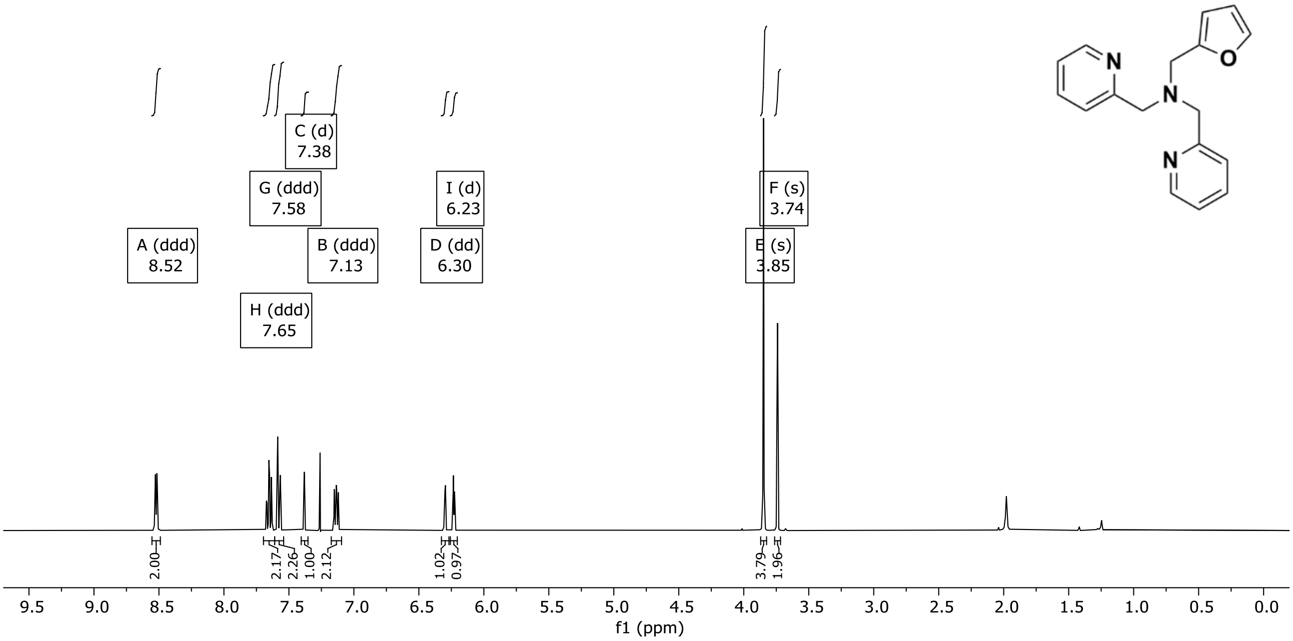

Figure S2. ^{1}H NMR spectrum of *N*-(Furan-2-ylmethyl)-*N*-[bis(2-pyridyl)methyl]amine (fubmpa) in $CDCl_3$.

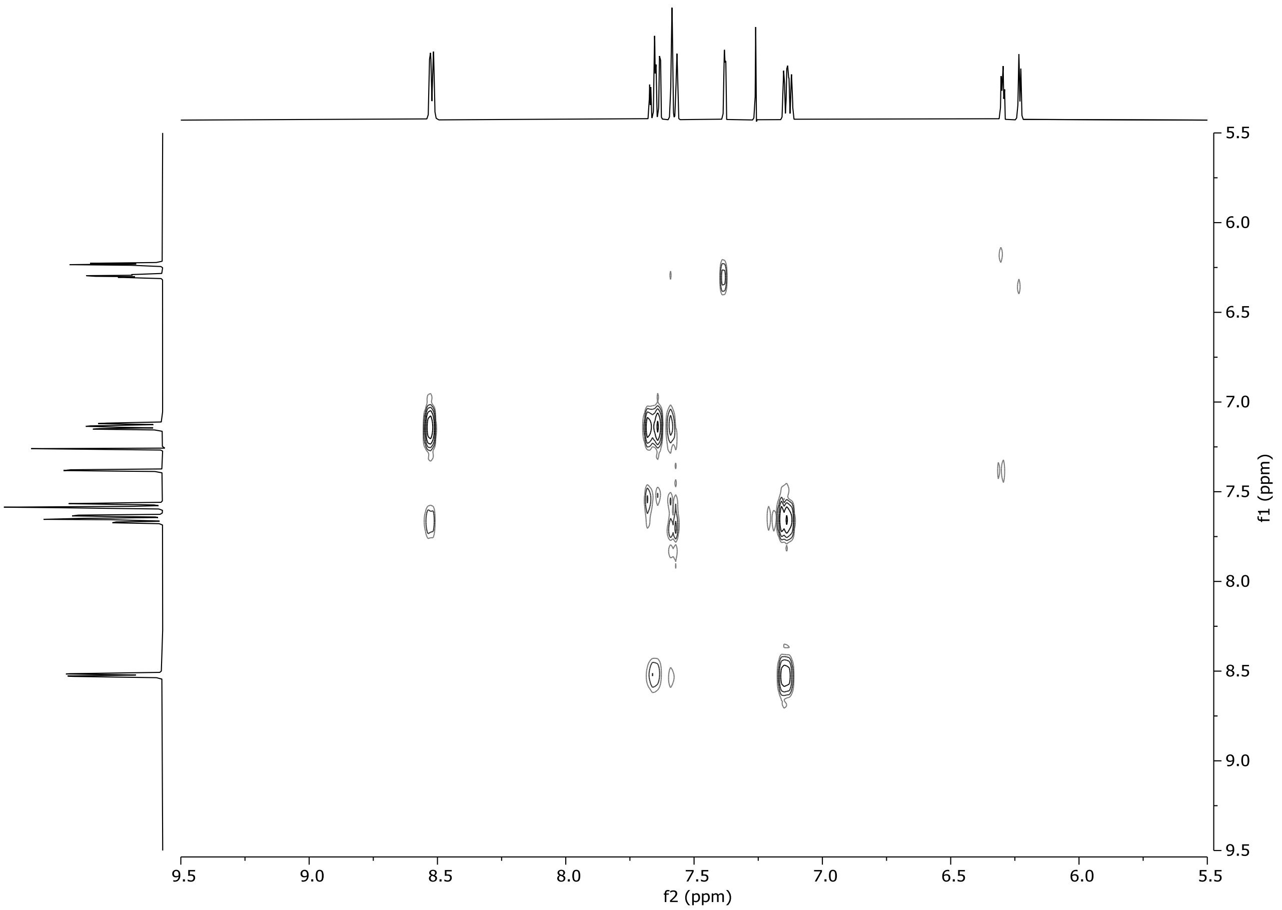

Figure S3. ^{1}H-^{1}H COSY NMR spectrum of *N*-(Furan-2-ylmethyl)-*N*-[bis(2-pyridyl)methyl]amine (fubmpa) in $CDCl_3$.

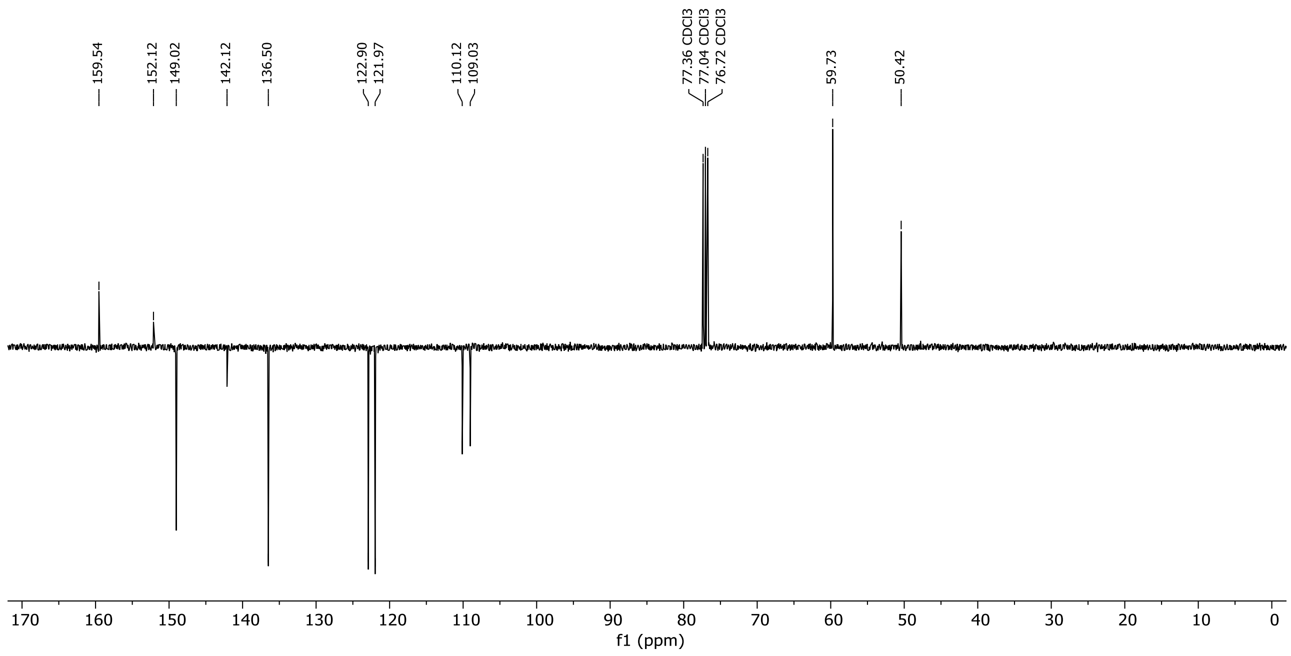

Figure S4. ^{13}C NMR spectrum of *N*-(Furan-2-ylmethyl)-*N*-[bis(2-pyridyl)methyl]amine (fubmpa) in $CDCl_3$.

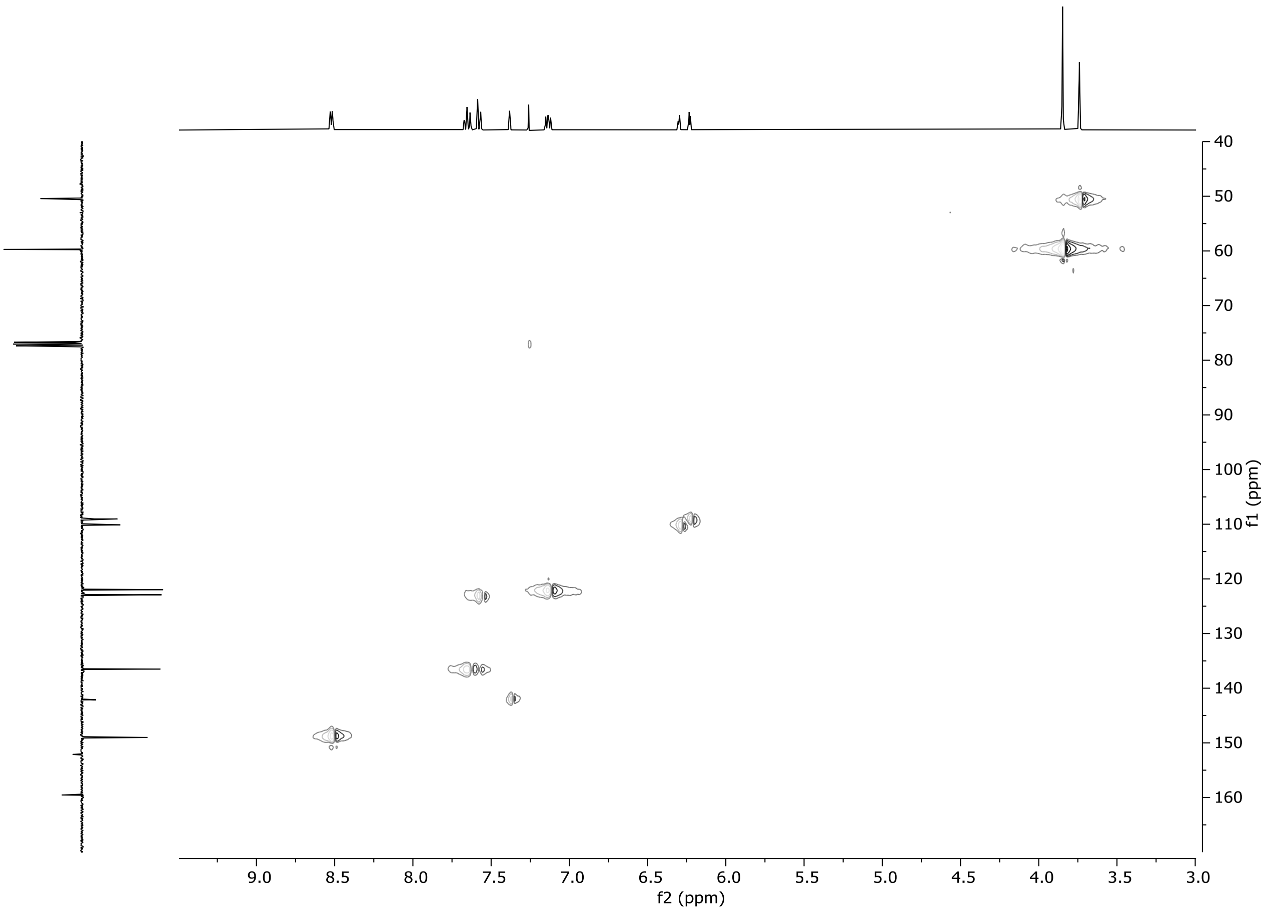

Figure S5. 2D HSQC spectrum of *N*-(Furan-2-ylmethyl)-*N*-[bis(2-pyridyl)methyl]amine (fubmpa) in $CDCl_3$.

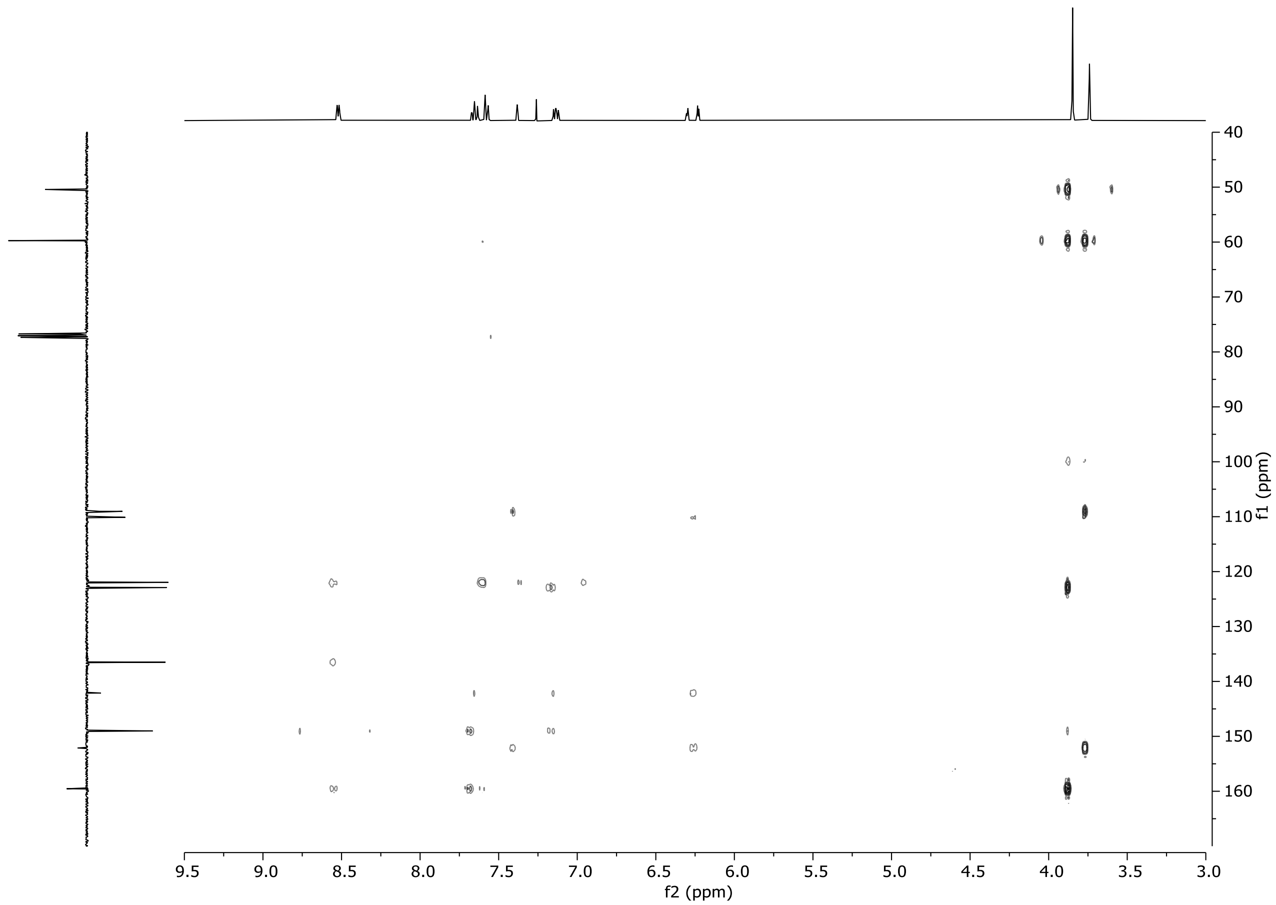

Figure S6. 2D HMBC spectrum of *N*-(Furan-2-ylmethyl)-*N*-[bis(2-pyridyl)methyl]amine (fubmpa) in $CDCl_3$.

S3. Crystallography data of Cu-fubmpa

All reflection intensities were measured at 110(2) K using a SuperNova diffractometer (equipped with Atlas detector) with Mo *K*α radiation (λ = 0.71073 Å) under the program CrysAlisPro (Version CrysAlisPro 1.171.39.29c, Rigaku OD, 2017). The same program was used to refine the cell dimensions and for data reduction. The structure was solved with the program SHELXS-2014/7 [3] and was refined on F^2 with SHELXL-2014/7.[3] Numerical absorption correction based on gaussian integration over a multifaceted crystal model was applied using CrysAlisPro. The temperature of the data collection was controlled using the system Cryojet (manufactured by Oxford Instruments). The H atoms were placed at calculated positions (unless otherwise specified) using the instructions AFIX 23 or AFIX 43 with isotropic displacement parameters having values 1.2 U_{eq} of the attached C atoms. The H atoms attached to O1W (coordinated water molecule) and O2W (lattice solvent molecule) were found from difference Fourier maps, and the O–H and H…H distances were refined pseudo freely using the DFIX instructions to keep their values within acceptable ranges. The structure is partly disordered. Both triflate anions are found to be disordered over two orientations. The occupancy factors of the major components of the disorder refine to 0.75(2) and 0.58(2). Bond lengths and angles of Cu-fubmpa are given in Table S1.

**Crystal data**	
Chemical formula	$C_{19}H_{19}CuF_6N_3O_8S_2\cdot H_2O$
M_r	677.05
Crystal system, space group	Monoclinic, $P2_1/c$
Temperature (K)	110
a, *b*, *c* (Å)	16.0028 (5), 9.8723 (3), 16.8915 (5)
β (°)	100.870 (3)

V (Å^3)	2620.71 (14)
Z	4
Radiation type	Mo $K\alpha$
μ (mm^{-1})	1.09
Crystal size (mm)	0.42 × 0.13 × 0.04

**Data collection**	
Diffractometer	SuperNova, Dual, Cu at zero, Atlas
Absorption correction	Gaussian<br>*CrysAlis PRO* 1.171.39.29c (Rigaku Oxford Diffraction, 2017) Numerical absorption correction based on gaussian integration over a multifaceted crystal model. Empirical absorption correction using spherical harmonics, implemented in SCALE3 ABSPACK scaling algorithm.
T_{min}, T_{max}	0.463, 1.000
No. of measured, independent and observed [$I > 2\sigma(I)$] reflections	18658, 6012, 4878
R_{int}	0.032
$(\sin \theta/\lambda)_{max}$ (Å^{-1})	0.650

**Refinement**	
$R[F^2 > 2\sigma(F^2)]$, $wR(F^2)$, S	0.035, 0.082, 1.04
No. of reflections	6012
No. of parameters	483
No. of restraints	501
H-atom treatment	H atoms treated by a mixture of independent and constrained refinement
$\Delta\rho_{max}$, $\Delta\rho_{min}$ (e Å^{-3})	0.42, -0.37

Table S1. Selected bond distances and bond angles of the crystal structure of Cu-fubmpa

Bond	Distance (Å)	Bond sequence	Angle (Å)	Bond sequence	Angle (Å)
**Cu1–N1**	1.9752(19)	**N1–Cu1–N2**	83.65(7)	**N2–Cu1–O1W**	168.08(7)
**Cu1–N2**	2.0369(18)	**N1–Cu1–N3**	166.31(8)	**N3–Cu1–O2**	90.89(7)
**Cu1–N3**	1.9615(19)	**N1–Cu1–O1W**	98.02(7)	**N3–Cu1–O1W**	93.61(8)
**Cu1–O1W**	1.9732(17)	**N1–Cu1–O2**	96.87(6)	**O2–Cu1–O1W**	87.14(6)
**Cu1–O2**	2.3749(15)	**N2–Cu1–N3**	83.50(7)		
**Cu1–O5**	2.665(2)	**N2–Cu1–O2**	104.42(6)		

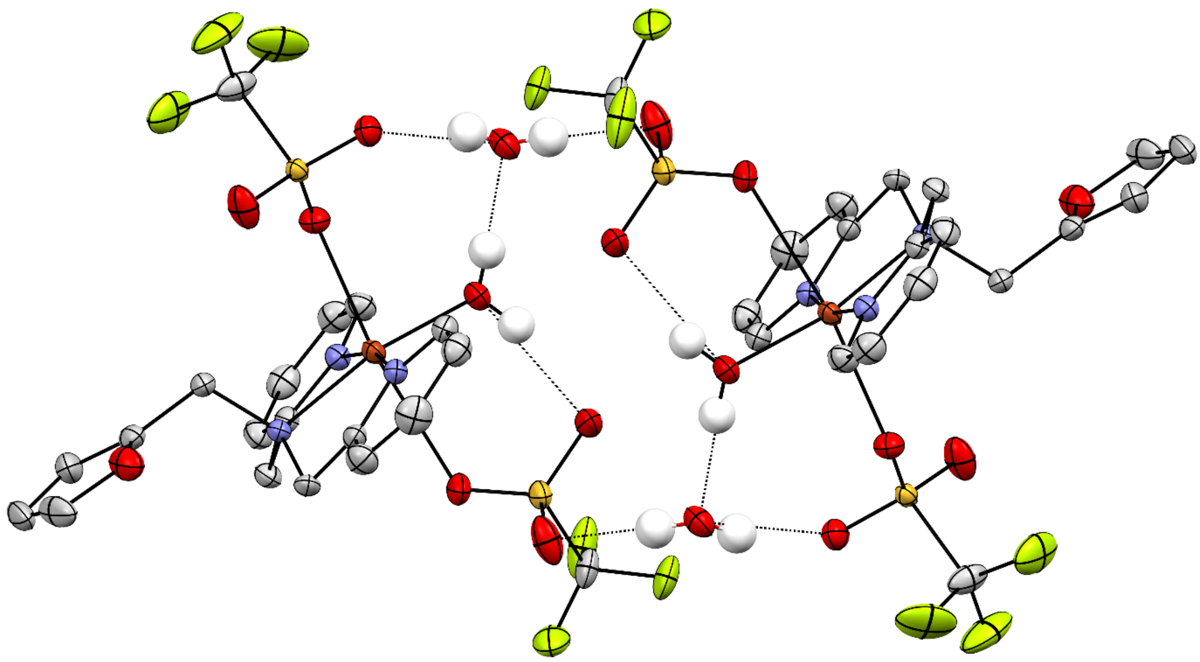

Figure S7 Displacement ellipsoid plot (50% probability level) of Cu-fubmpa at 110(2) K, showing hydrogen bond interactions between Cu-fubmpa and lattice water solvent molecules. Other H atoms and disorder are omitted for clarity.

S4 Data obtained from Cyclic Voltammetry

To determine the diffusion coefficient (D) for these complexes, CVs were measured at different scan rates, varying from 10 to 500 mV s^{-1}, for each complex and are shown in Figure S8. In the same figure, the corresponding Randles-Sevcik plots show that good linearity ($R^2 > 0.99$) is achieved for the i_{pc} and i_{pa} as a function of the square root of the scan rate. This indicates that the complexes behave as diffusive homogenous species near the electrode under inert conditions, and potential deposition of the complex on the electrode surface does not play a significant role. By applying the Randles-Sevcik equation (Eq. 1), the diffusion coefficients of the Cu^{II} species were determined from the i_{pc} values (Table 1, main text).

$$i_p = 0.446nFSC_{cat}^{0}\sqrt{\frac{Fv}{RT}D_{cat}} \qquad \text{(S1)}$$

The redox couples of all the complexes seem to be fully reversible, but analysis of the peak-to-peak separation (ΔE_p) shows a small deviation from the ideal 59 mV peak-to-peak separation for a fully reversible system, averaging a ΔE_p increase of 10 mV at a 100 mV s^{-1} scan rate. To investigate whether this is the case over a larger range of scan rates, Laviron plots of the oxidative (E_{pa}) and reductive (E_{pc}) peak potentials were constructed (Figure S9). An increase of the ΔE_p with increasing scan rate is observed for all three complexes, especially at scan rates above 100 mV s^{-1}. This increase is largely caused by a shift of the E_{pa} of the respective complexes towards higher potentials, while the E_{pc} remain stable or show much smaller shifts. As a result, the $E_{1/2}$, which is defined as the midway potential between the E_{pc} and E_{pa}, is also affected. For Cu-fubmpa and Cu-bpmpa, this leads to an apparent positive shift of the $E_{1/2}$ at scan rates above 100 mV s^{-1} (Figure S10). Cu-pmea sees both the E_{pa} and E_{pc} equally shift towards higher and lower potentials, respectively, leading to a stable $E_{1/2}$ as a function of scan rate. The increase in peak-to-peak separation resulting from a shifting E_{pa} may point to slower electron transfer rate for the oxidation of the Cu^{I} species,[4] although the effect is marginal with only an increase of 15–20 mV observed for the ΔE_p.

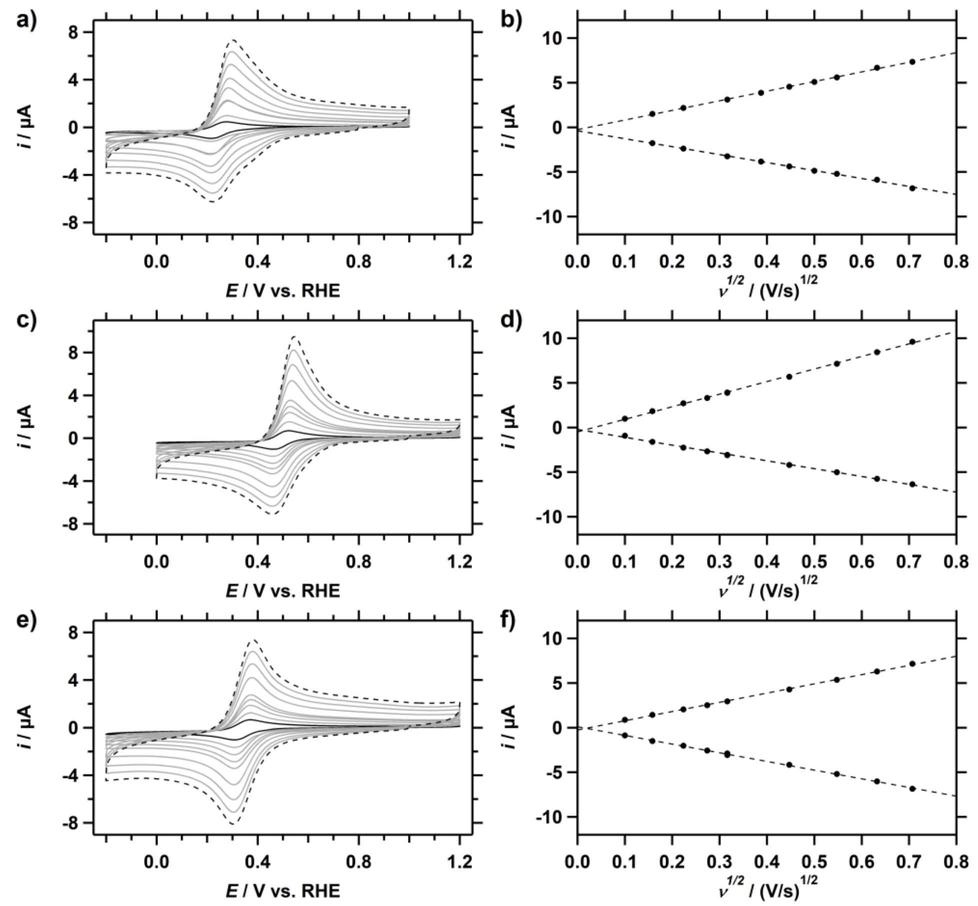

Figure S8. CVs of Cu-fubmpa **(a)**, Cu-bpmpa **(b)**, and Cu-pmea **(c)** over a range of scan rates from 10 mV s^{-1} (solid black) to 500 mV s^{-1} (dashed). A concentration of 0.3 mM was used for each catalyst. Conditions: pH 7 PB ([PO_4] = 100 mM), 293 K, 0.0707 cm^2 electrode surface area.

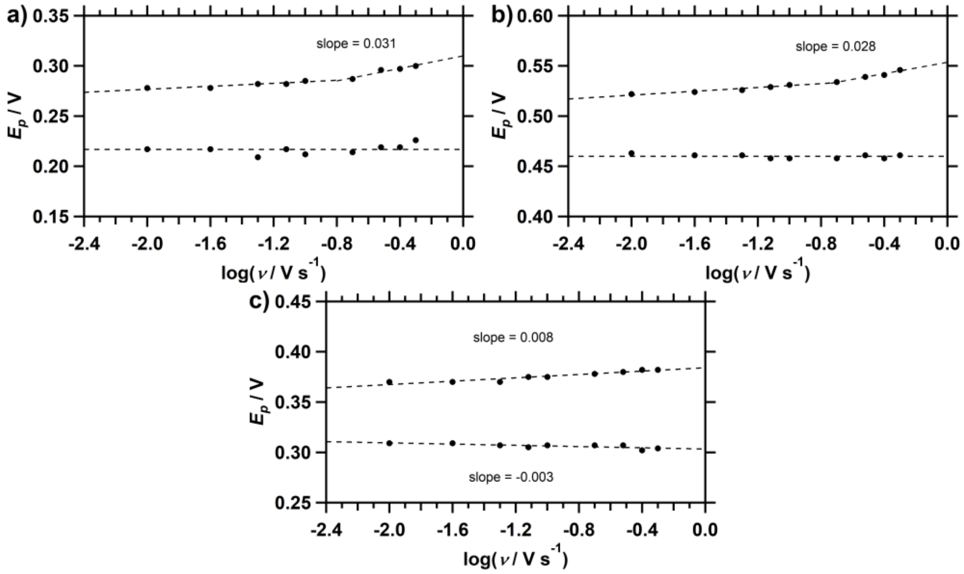

Figure S9. Laviron plots showing the peak potentials as a function of the logarithm of the scan rate for Cu-fubmpa **(a)**, Cu-bpmpa **(b)**, and Cu-pmea **(c).** A concentration of 0.3 mM was used for each catalyst

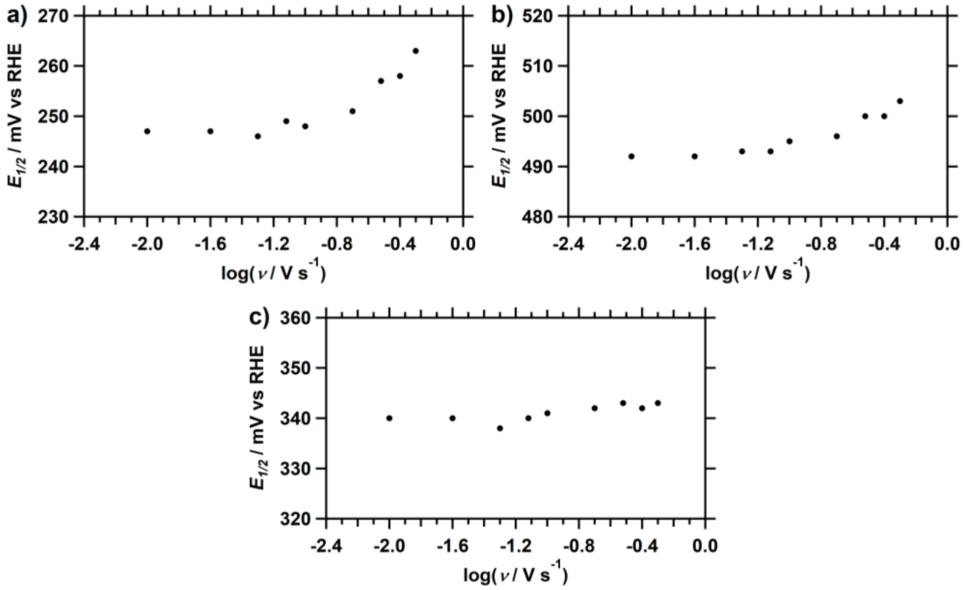

Figure S10. Plot of redox half-wave potential as a function of scan rate for Cu-fubmpa **(a)**, Cu-bpmpa **(b)**, and Cu-pmea **(c)**.

S5. Catalytic performance of Cu-bpmpa

Cu-bpmpa shows an onset that is near the equilibrium potential of the O_2/H_2O_2 couple, albeit with a very low current. Additionally, a second, larger catalytic wave is observed at a much lower potential. The half-wave potential of the first catalytic wave of Cu-bpmpa is the same as the equilibrium potential of the redox couple in the absence of O_2. This equivalence, where $E_{1/2}$ is equal to the half-wave potential of the catalytic wave ($E_{cat/2}$), is expected for a system that is not limited in substrate. This behaviour is not observed for the $E_{cat/2}$ of Cu-fubmpa (+90 mV) or Cu-pmea (+25 mV) at catalyst concentration of 0.3 mM. However, for Cu-bpmpa no limiting current plateau is maintained and the catalytic current increases again at potentials below 0.3 V vs. RHE. In this potential window, the background current generated by the glassy carbon electrode needs to be accounted for, as GC readily reduces O_2 to H_2O_2 below 0.3 V. This could be the main contributing factor to the catalytic current observed in the lower potential region. Background correction on the catalytic waves was performed by subtracting the background current of the GC electrode from the current measured in presence of catalyst (Figure S11), which shows that the second catalytic wave is much less prominent than in Figure 4 (main text), but is still present and reaches a peak catalytic current i_{cat} at 0.1 V vs. RHE.

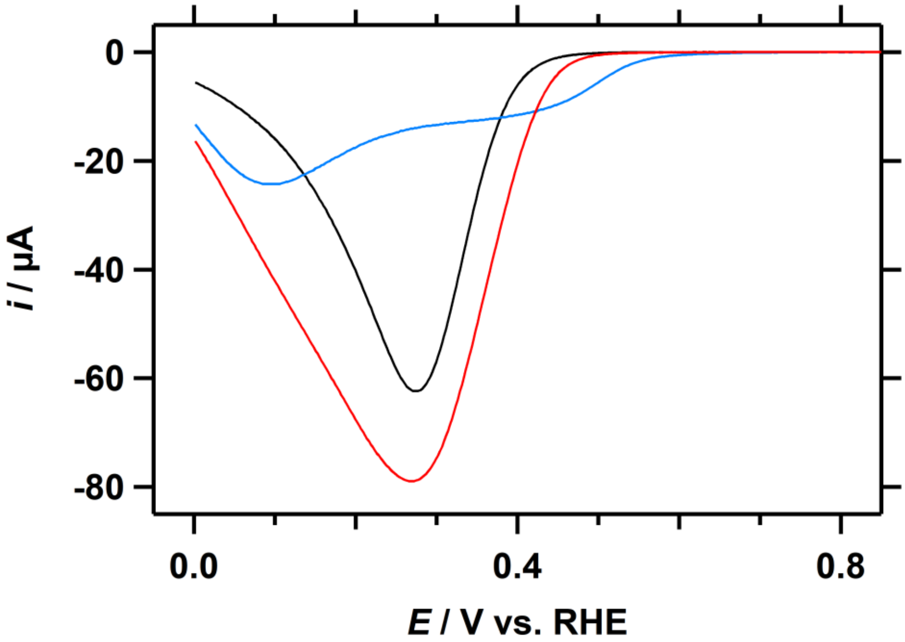

Figure S11. Background corrected LSV of the ORR. LSV of Cu-fubmpa (black), Cu-pmea (red), and Cu-bpmpa (blue), under 1 atm O_2. A catalyst concentration of 0.3 mM was used for each complex. Conditions: pH 7 PB ($[PO_4]$ = 100 mM), 293 K, 100 mV s^{-1} scan rate, 0.0707 cm^2 electrode surface area.

S6. Determination of the catalytic rate

S6.1. Foot-of-the-wave analysis

As discussed in the main text, foot-of-the-wave analysis (FOWA) was carried out to obtain the maximum turnover frequency (TOF_{max}) for every catalyst for both the ORR and HPRR. The FOWA graphs of Cu-fubmpa are shown in Figure 12 and 15, for Cu-pmea in Figure 13 and 16, and for Cu-bpmpa inFigure 14 and 17. For all catalysts the catalytic CVs are shown from which the TOF_{max} values were extracted. Plots of i_c/i_p vs $1/(1+\exp((E-E_{1/2})f)$ are shown, where i_c is the catalytic current measured in the presence of catalyst and substrate at the applied potential E and i_p is the peak current of the Cu^{II} reduction in absence of substrate. The TOF_{max} was determine from the slope of a linear fit in the region where $i_c/i_{redox} \geq 2$ and i_c/i_p of at least 1.6, where i_{redox} is the current measured in presence of catalyst, but in absence of substrate. In all cases, the R^2 of this linear fit was at least 0.98, except for the HPRR measurements Cu-fubmpa, where $R^2 \geq 0.95$ was found. All TOF_{max} values, including the average values, are shown in Table 2 and 3.

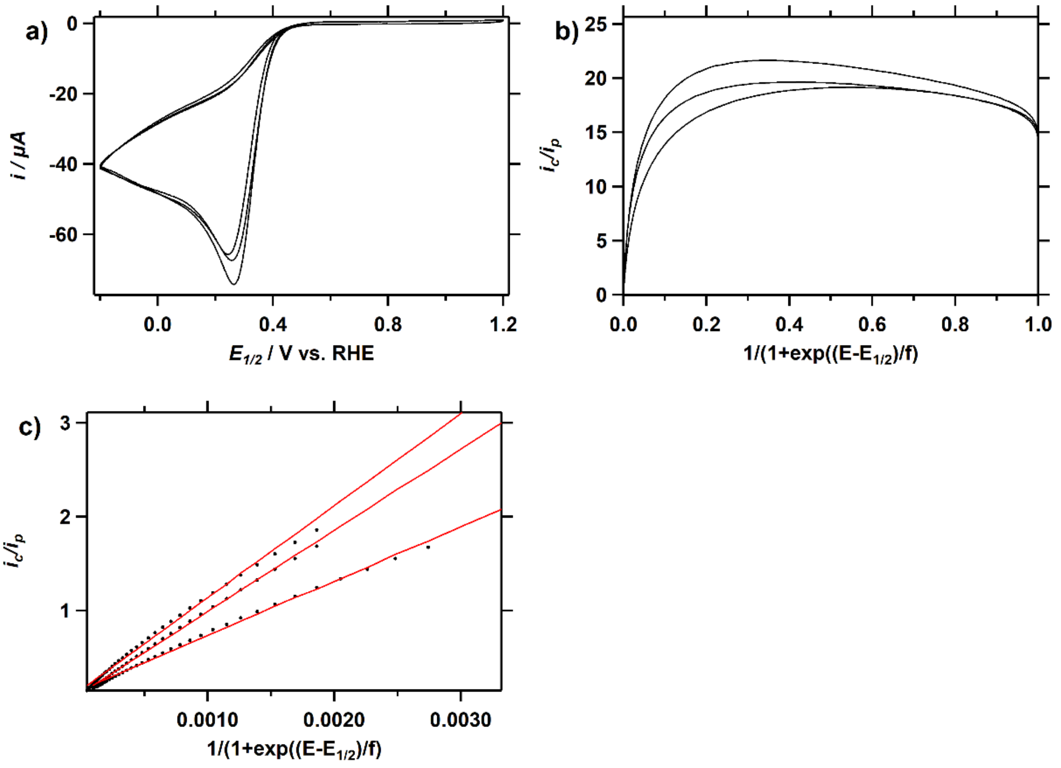

Figure S12. ORR CVs in presence of 0.3 mM Cu-fubmpa (a), FOWA of the ORR, where f = F/RT (b). Corresponding fits of the linear regions of the FOWA, $R^2 \geq 0.98$. Conditions: pH 7 PB ([PO_4] = 100 mM), 293 K, 100 mV s^{-1} scan rate.

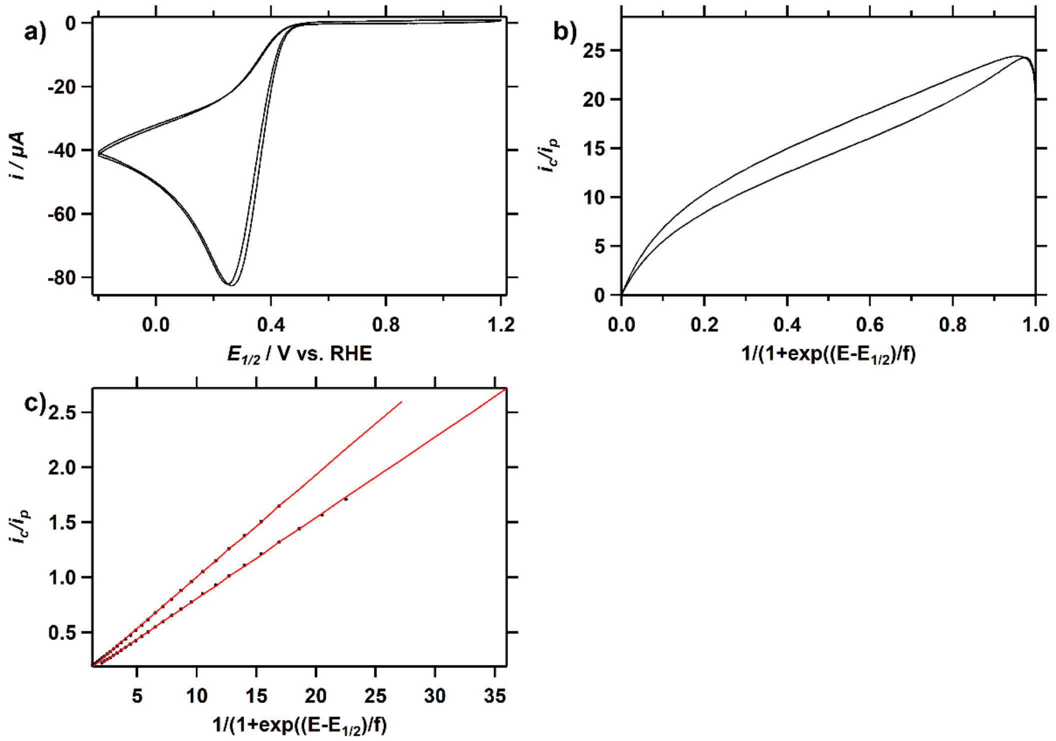

Figure S13. ORR CVs in presence of 0.3 mM Cu-pmea (a), FOWA of the ORR, where f = F/RT (b). Corresponding fits of the linear regions of the FOWA, $R^2 \geq 0.98$. Conditions: pH 7 PB ($[PO_4]$ = 100 mM), 293 K, 100 mV s^{-1} scan rate.

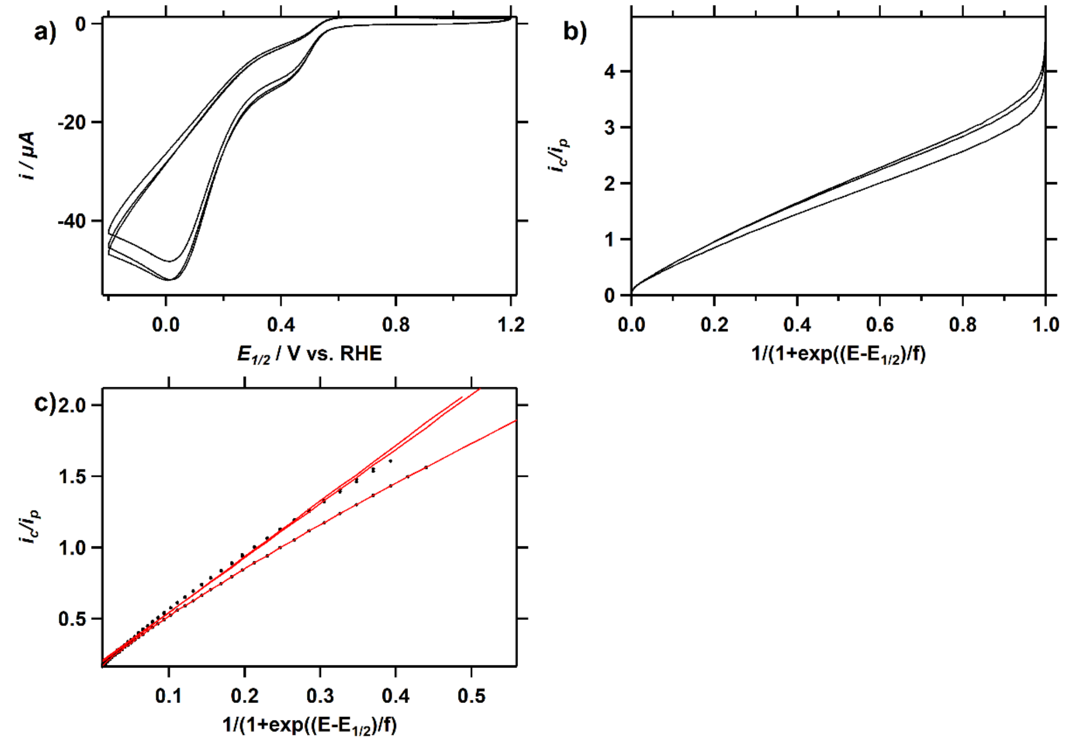

Figure S14. ORR CVs in presence of 0.3 mM Cu-bpmpa (a), FOWA of the ORR, where f = F/RT (b). Corresponding fits of the linear regions of the FOWA, $R^2 \geq 0.98$. Conditions: pH 7 PB ($[PO_4]$ = 100 mM), 293 K, 100 mV s^{-1} scan rate.

Table S2. Overview of the TOF_{max} values determined for the ORR by Cu-fubmpa, Cu-pmea, and Cu-bpmpa in CV experiments, in a pH 7 phosphate buffer under 1 atm O_2. [a] Determined from data from ref 6.

Complex	$TOF_{max,ORR}$ (s^{-1})			$TOF_{max,ORR}$ Average (s^{-1})
Cu-tmpa [a]				$1.8\times10^{6} \pm 0.6\times10^{6}$
Cu-fubmpa	1.48×10^{5}	1.89×10^{5}	6.61×10^{4}	$1.3\times10^{5} \pm 0.3\times10^{5}$
Cu-pmea	1.71×10^{3}	1.07×10^{3}	*n.d.*	$1.4\times10^{3} \pm 0.2\times10^{3}$
Cu-bpmpa	7.60×10^{-1}	7.21×10^{-1}	5.35×10^{-1}	0.7 ± 0.06

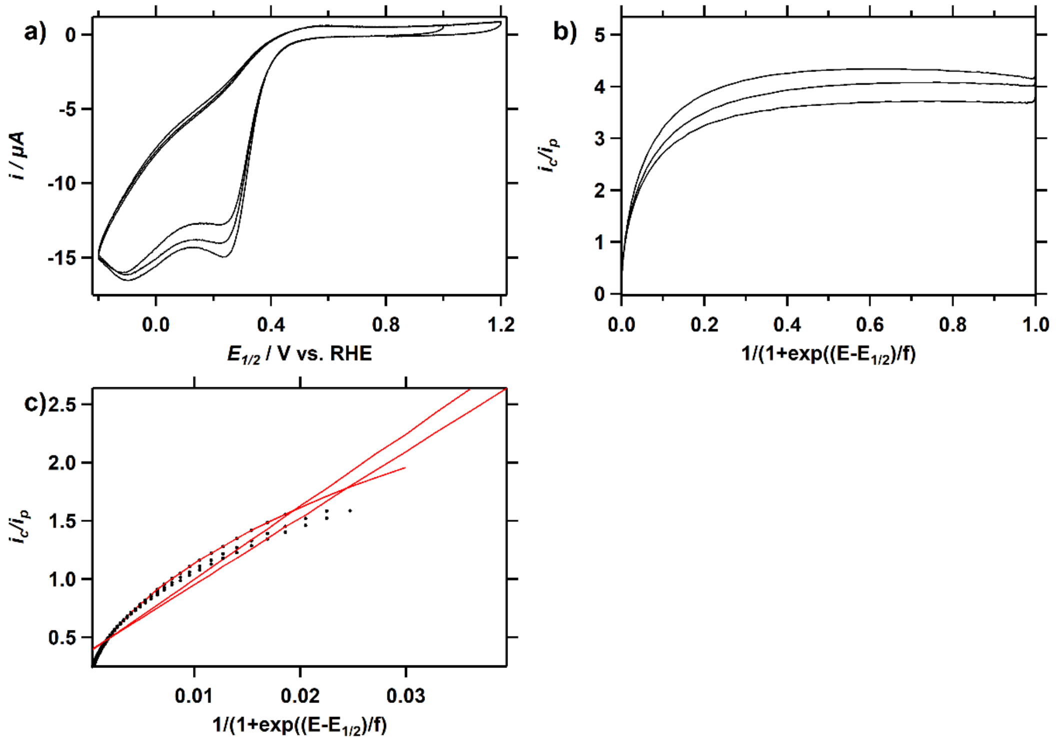

Figure S15. HPRR CVs in presence of 0.3 mM Cu-fubmpa and 1.1 mM H_2O_2 (a), FOWA of the HPRR, where f = F/RT (b). Corresponding fits of the linear regions of the FOWA, $R^2 \geq 0.95$. Conditions: pH 7 PB ([PO_4] = 100 mM), 293 K, 100 mV s^{-1} scan rate.

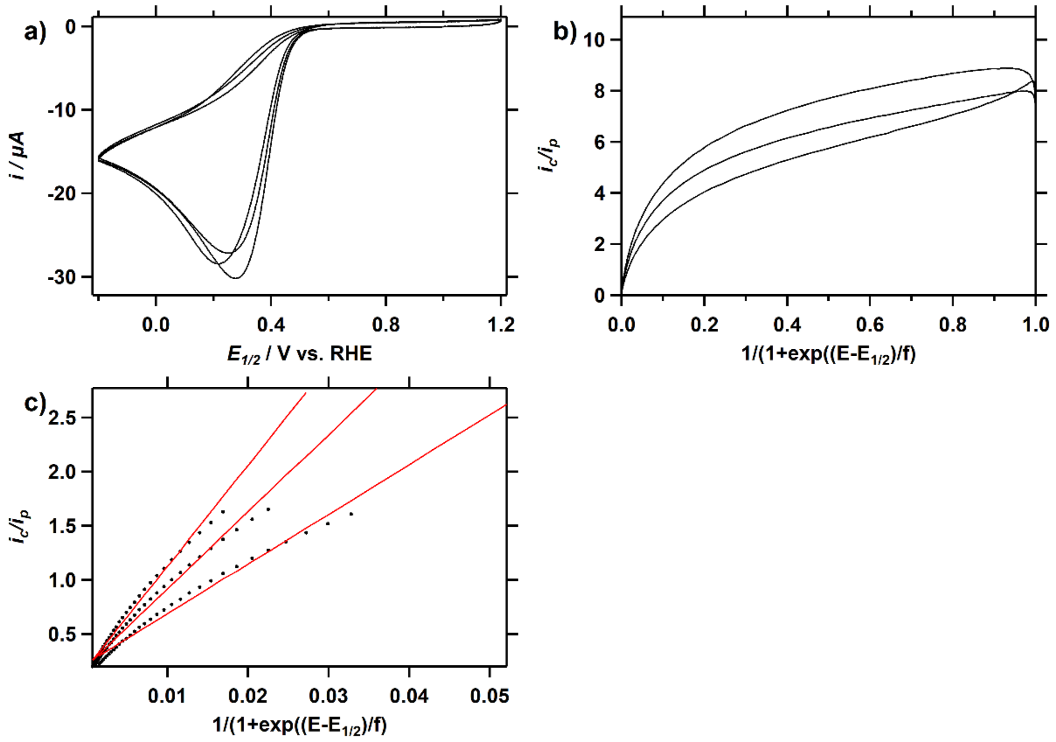

Figure S16. HPRR CVs in presence of 0.3 mM Cu-pmea and 1.1 mM H_2O_2 (a), FOWA of the HPRR, where f = F/RT (b). Corresponding fits of the linear regions of the FOWA, $R^2 \geq 0.98$. Conditions: pH 7 PB ([PO_4] = 100 mM), 293 K, 100 mV s^{-1} scan rate.

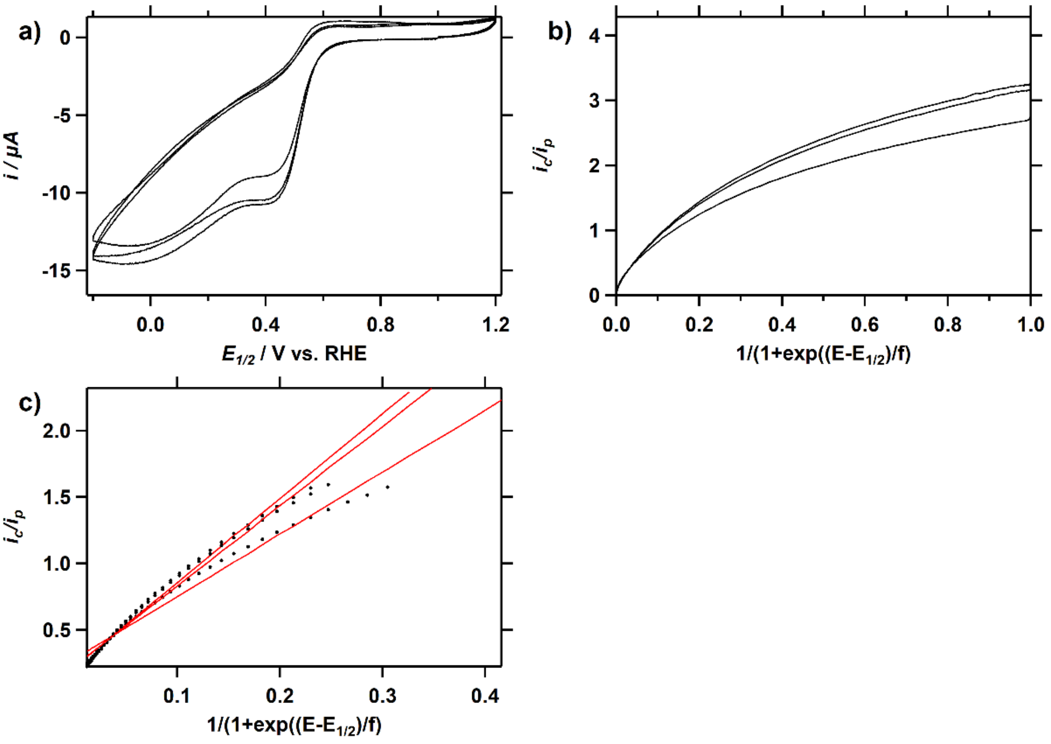

Figure S17. HPRR CVs in presence of 0.3 mM Cu-bpmpa and 1.1 mM H_2O_2 (a), FOWA of the HPRR, where f = F/RT (b). Corresponding fits of the linear regions of the FOWA, $R^2 \geq 0.98$. Conditions: pH 7 PB ([PO_4] = 100 mM), 293 K, 100 mV s^{-1} scan rate.

Table S3. Overview of the TOF_{max} values determined for the HPRR by Cu-fubmpa, Cu-pmea, and Cu-bpmpa in CV experiments, in a pH 7 phosphate buffer under 1 atm Ar and in presence of 1.1 mM H_2O_2. [a] Determined from data from ref 7.

Complex	$TOF_{max,HPRR}$ (s^{-1})			$TOF_{max,HPRR}$ Average (s^{-1})
Cu-tmpa [a]				$2.1\times10^5 \pm 0.1\times10^5$
Cu-fubmpa	1.06×10^3	7.79×10^2	6.49×10^2	$8.3\times10^2 \pm 1.0\times10^2$
Cu-pmea	1.63×10^3	9.50×10^2	4.14×10^2	$1.0\times10^3 \pm 2.9\times10^2$
Cu-bpmpa	4.32	7.91	7.11	6.4 ± 0.9

S6.2. Current enhancement method

We have used FOWA to determine the TOFs for all catalysts discussing in this manuscript as satisfactory results are obtained using this method while its use simultaneously avoids complications due to catalyst modification under catalytic conditions. In addition we have used CE to verify that the same active species are operating at the peak of the catalytic wave as at the foot of the catalytic wave. The CE experiments outlined below illustrate that FOWA and CE data correlate well for Cu-tmpa and Cu-fubmpa, but that in case of Cu-pmea and Cu-bpmpa a more active species is formed at the peak of the catalytic wave compared to the foot of the catalytic wave. This confirms that FOWA is the appropriate technique to compare the $E_{1/2}$ values of all catalysts with a rate constant, and that the RRDE data far beyond the onset of ORR should be taken with a grain of salt.

The current enhancement method is an alternative to the FOWA (discussed in the main text) to determine the catalytic performance by direct determination using the catalytic current enhancement derived from the i_{pc} of the catalyst and the i_{cat}.[5] Ideally, this should be done under reliable kinetic conditions, such that substrate diffusion is not the main limiting factor during the catalytic reaction. In the case of a highly active ORR catalyst, this requires measurement of the current enhancement at low catalyst concentration. However, the determination of the i_{pc} at low catalyst concentration is complicated by the relatively much larger double layer current of the electrode. Therefore, the i_{pc} is derived from the Randles-Sevcik equation (Eq. S1), using the calculated diffusion coefficient of the catalyst and the catalyst concentration. The i_{cat} values were obtained from background-corrected LSVs measured at several catalyst concentrations in the range of 1 to 30 μM, depending on the catalyst, under 1 atm O_2 (see Figure S18). The k_{obs} were derived from the current enhancement (i_{cat}/i_p) using Eq. S2, in the concentration range where a linear dependency of the i_{cat} on the catalyst concentration was observed. Eq. S2 and Eq. 1 (from the main text) are equal for the case when the

applied potential E is lower than the $E_{1/2}$, and the $(1 + exp[F/RT(E - E_{1/2})])^{-1}$ term goes to unity, and a maximum catalytic current is reached, where $i_c = i_{cat}$.

$$\frac{i_{cat}}{i_{pc}} = 2.24n\sqrt{\frac{RT}{Fv}k_{obs}} \qquad \text{(S2)}$$

CE analysis resulted in ORR k_{obs} of $2.0\times10^3 \pm 0.6\times10^3$ s^{-1} for Cu-fubmpa, $2.0\times10^4 \pm 0.2\times10^4$ s^{-1} for Cu-pmea, and $0.7\times10^3 \pm 0.1\times10^3$ s^{-1} for Cu-bpmpa, all of which are lower than the k_{obs} of Cu-tmpa (2.0×10^5) which was determined using the same method.[6] Comparing the k_{obs} to the previously determined TOF_{max}, a large difference of three orders of magnitude is observed for the rate constants of Cu-bpmpa. As mentioned above, the TOF_{max} of this complex describes the catalytic rate constant associated with the first catalytic wave, while the k_{obs} was determined from the peak catalytic current around 0.13 V at low catalyst concentration (Figure S18e), which corresponds to the second catalytic wave observed. A catalytic current associated with the first catalytic wave cannot be observed at these low catalyst concentrations (Figure S18e). However, if Eq S2 is applied to the smaller first catalytic wave in the presence of 0.3 mM Cu-bpmpa and 1 atm O_2 (Figure S19), a k_{obs} of 0.6 s^{-1} is obtained. Here, the catalytic peak current of the first catalytic wave (E_{cat} = 0.4 V vs. RHE) was used as i_{cat}, while i_{pc} was obtained from the redox couple of the complex under inert atmosphere. This k_{obs} value (0.6 s^{-1}) corresponds closely to the FOWA-derived TOF_{max} of the ORR (0.7 s^{-1}) under the same catalytic conditions (0.3 mM Cu-bpmpa, 1 atm O_2). The k_{obs} of Cu-pmea was found to be slightly less than two orders of magnitude higher than the TOF_{max}. In general, for the same catalytic reaction and catalytic species, the k_{obs} obtained from the peak catalytic current is lower than the ideal TOF_{max}. While substrate depletion has been reduced by increasing the ratio between O_2 and the catalyst under the conditions where the k_{obs} for Cu-pmea was determined, other processes may still negatively affect the catalytic reaction and thus decrease the measured k_{obs}. This holds true for the other two catalysts described here, including Cu-tmpa.[6] The contradictory results for Cu-pmea cannot be explained by a potential erroneous choice of n_{cat}, (although we assume that n=2 is most

appropriate due to the accumulation of hydrogen peroxide,[6] rather than n=4). Additionally, at low catalyst concentration the half-wave potential $E_{cat/2}$ during ORR is equal to the $E_{1/2}$ of the catalyst, confirming that the species present under non-catalytic conditions is also responsible for the peak current under catalytic conditions and thus for the measured k_{obs}. Also, no overlapping catalytic peaks are observed during differential pulse voltammetry measurements under catalytic conditions (Figure S20), which would point to the presence of two different Cu-pmea complexes is present. Some Cu-species do easily stick to the carbon electrode surface,[8, 9] and especially at very low catalyst concentrations where the Cu^{II}/Cu^{I} redox couple is not clearly visible it remains difficult to account for the correct number of active sites. Hence, we do believe that the reaction rates obtained by FOWA represent a more meaningful picture in this context.

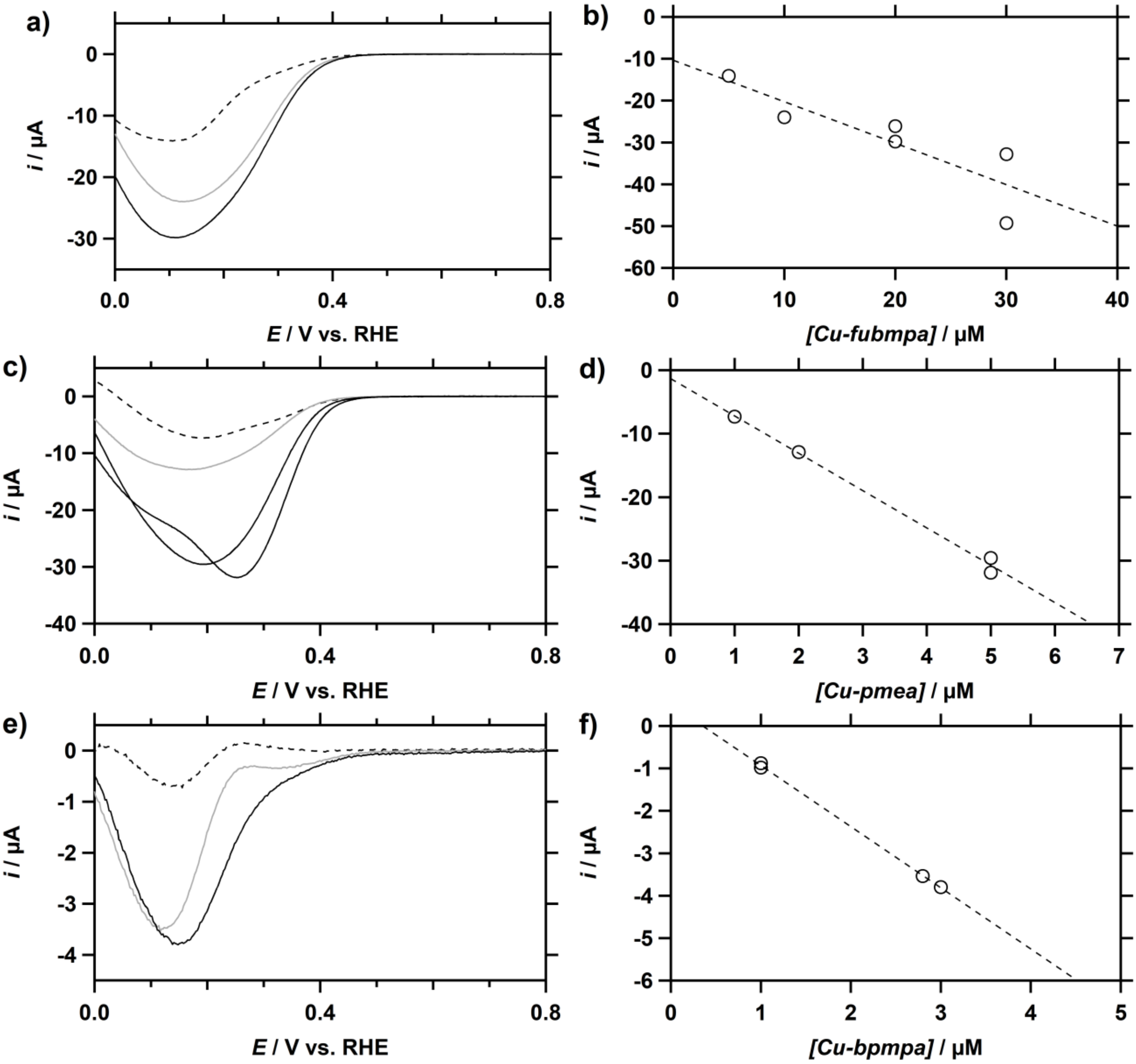

Figure S18. Catalytic ORR activity at low catalyst concentrations. Background-corrected LSV of-fubmpa **(a)**, Cu-pmea **(c)**, and Cu-bpmpa **(e)** at different catalyst concentrations in the presence of 1 atm O_2. On the right, the corresponding peak catalytic current obtained from the LSV of Cu-fubmpa **(b)**, Cu-pmea **(d)**, and Cu-bpmpa **(f)** as a function of catalyst concentration. Conditions: pH 7 PB ([PO_4] = 100 mM), 293 K, 100 mV s^{-1} scan rate, 0.0707 cm^2 electrode surface area.

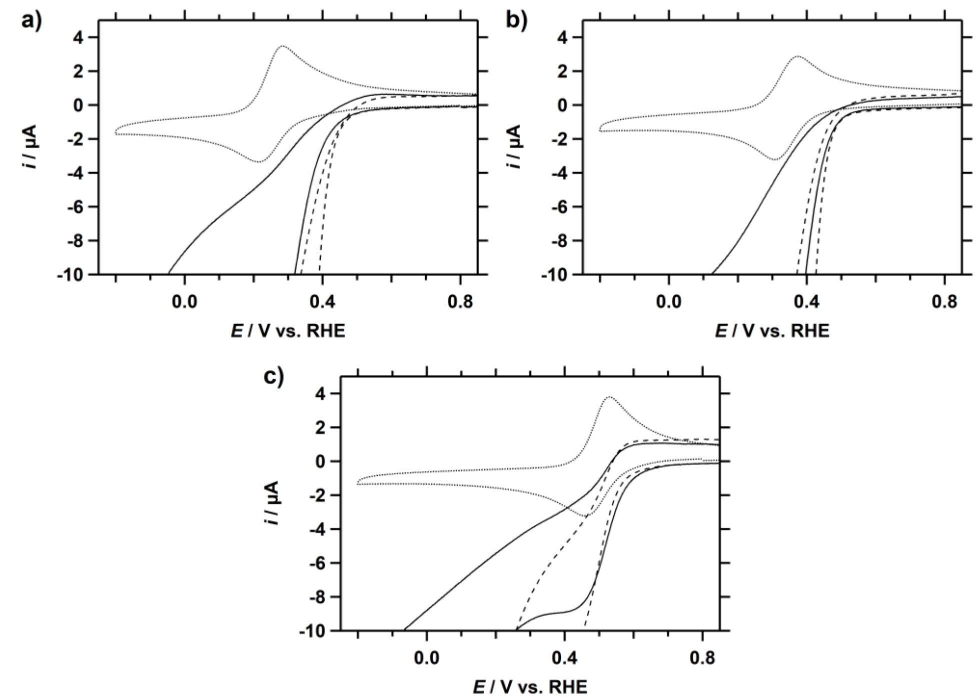

Figure S19. Comparison of onset ORR and HPRR. CVs of Cu-fubmpa **(a)**, Cu-pmea **(b)**, and Cu-bpmpa **(c)** in a PB pH 7 electrolyte solution under 1 atm Ar (dotted line), 1 atm O_2 (dashed line), or with 1.1 mM H_2O_2 under 1 atm Ar (solid line). For each catalyst, a concentration of 0.3 mM was used. Conditions: pH 7 PB ([PO_4] = 100 mM), 293 K, 100 mV s^{-1} scan rate, 0.0707 cm^2 electrode surface area.

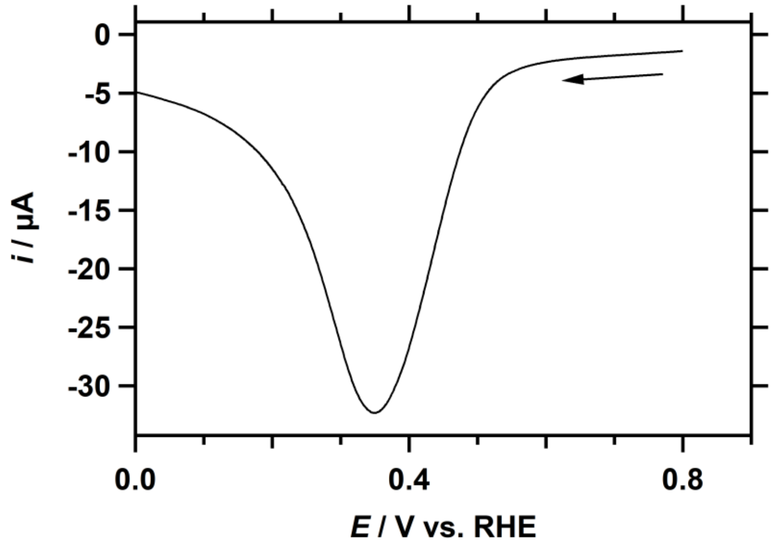

Figure S20. DPV of Cu-pmea (0.3 mM) in the presence of 1 atm. O_2. Conditions: pH 7 PB ([PO_4] = 100 mM), 293 K, 100 mV s^{-1} scan rate, 0.0707 cm^2 electrode surface area.

S7. Rotating Ring-Disk Electrochemistry measurements

Prior to every RRDE experiment the collection efficiency of the Pt ring was determined. This is important to be able to exactly quantify the H_2O_2 that is produced at the disk during the ORR. As GC converts O_2 only to H_2O_2 during the ORR, the collection efficiency of the RRDE setup can be determined by studying the activity of the blank electrode. To do so, a freshly polished electrode was used to record a CA measurement of 5 minutes with the disk set to -0.1 V vs. RHE and the ring set to 0.8 V vs. RHE. Next, the ring current was corrected for the double layer current recorded at 0.8 V vs. RHE (disk potential) prior to the experiment. The final collection efficiency was determined by dividing the ring current by the disk current, and averaged over the first 30-60 seconds of the experiment.

Catalytic RRDE CVs of Cu-fubmpa, Cu-bpmpa, and Cu-pmea were recorded in 0.1 M PB (Figure S21). For every catalyst, the RRDE experiment was repeated three times on a freshly polished electrode to make sure the results were reproducible. Next, the ring current of all measurements was divided by the collection efficiency of the Pt ring, which was determined prior to every experiment. In addition, the current at the ring was corrected for the background current measured in every experiment between 0.8 and 0.7 V vs. RHE. In this manner, the ring currents depicted in Figure S21, can be used to make a fair comparison between the different catalysts. In turn, Figure S21b shows one of the LSV measurements for all catalysts, compared to the previously reported results of Cu-tmpa.[6]

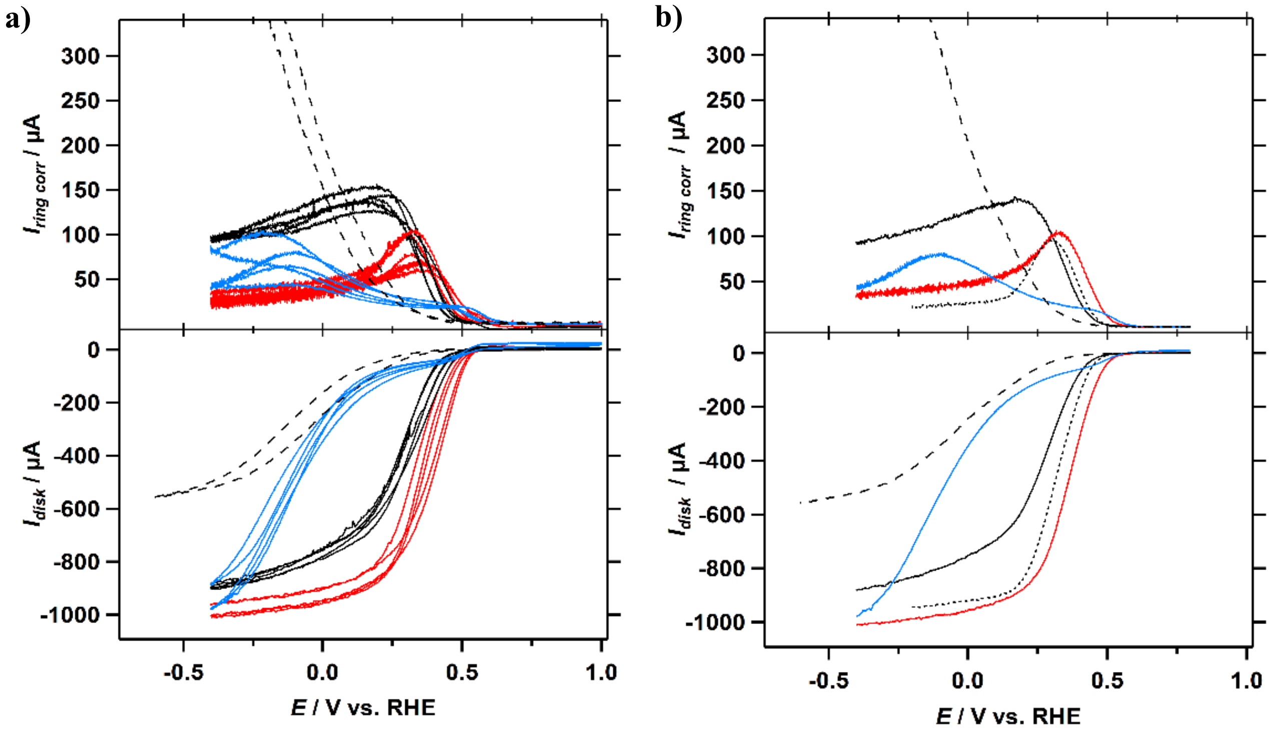

Figure S21. RRDE CV (left) and LSV (right) experiments of Cu-fubmpa (black), Cu-pmea (red), Cu-bpmpa (blue), and the GC electrode in absence of catalyst (dashed line), under 1 atm O_2. **(b)** shows LSV data for Cu-tmpa (dotted line) for reference, obtained from Ref 6. A catalyst concentration of 0.3 mM was used for each complex. Conditions: pH 7 PB ([PO_4] = 100 mM), 293 K, 50 mV s^{-1} scan rate, 0.196 cm^2 electrode surface area, 1600 RPM, Pt ring at 1.2 V vs. RHE.

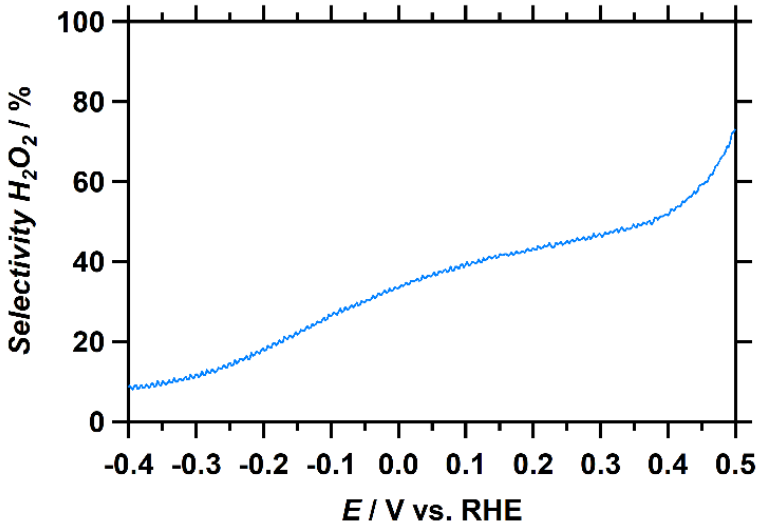

Figure S22. H_2O_2 selectivity of Cu-bpmpa determined from RRDE data. A catalyst concentration of 0.3 mM was used. Conditions: pH 7 PB ($[PO_4]$ = 100 mM), 293 K, 50 mV s^{-1} scan rate, 0.196 cm^2 electrode surface area, 1600 RPM, Pt ring at 1.2 V vs. RHE.

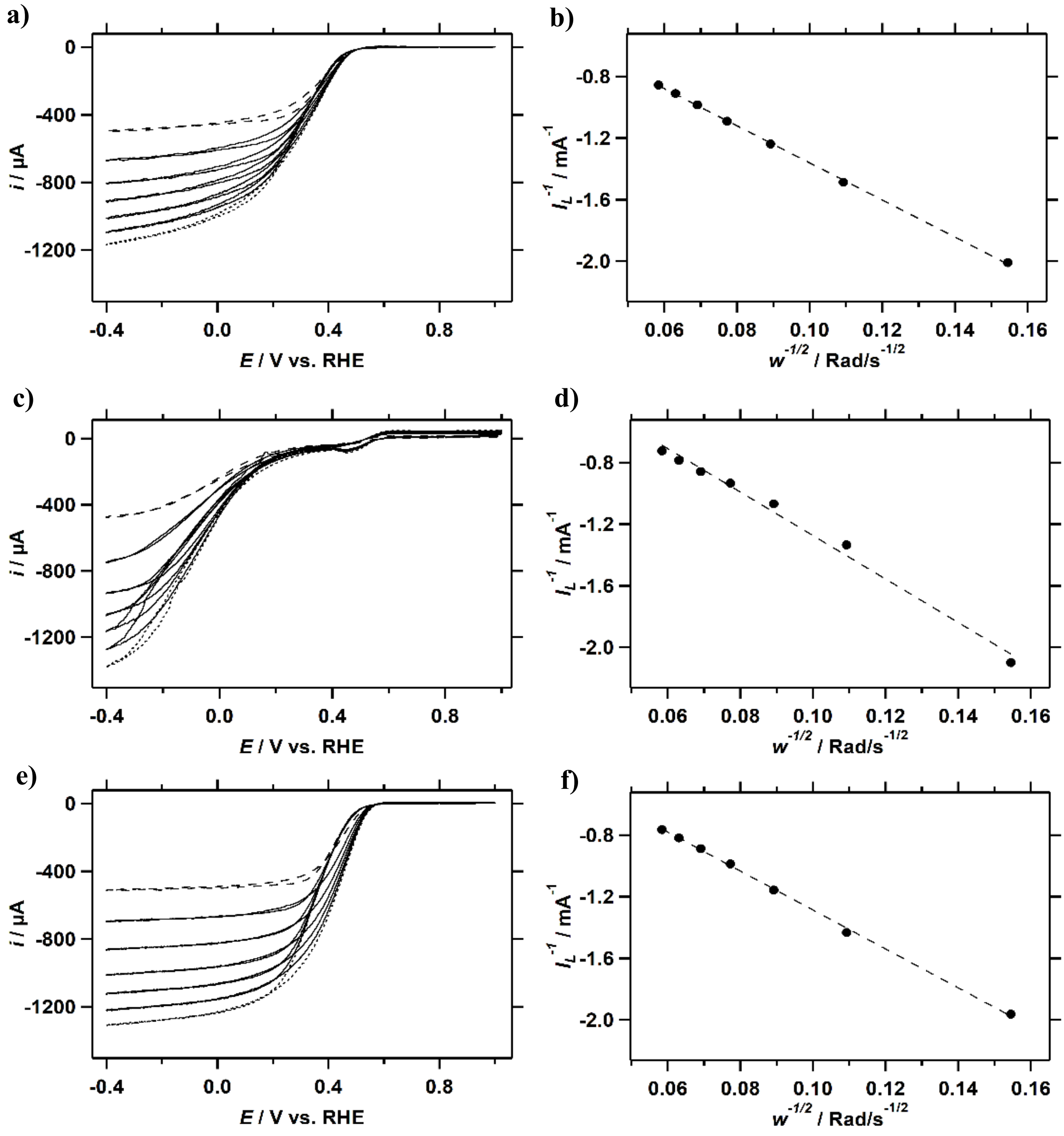

Figure S23. Koutecky-Levich plots. RDE CVs of Cu-fubmpa **(a)**, Cu-bpmpa **(c)**, and Cu-pmea **(e)** under 1 atm O_2 with varying rotation rates between 400 RPM (dashed line) and 2800 RPM (dotted line). Corresponding Koutecky-Levich plots derived from catalytic currents at -0.4 V vs. RHE for Cu-fubmpa **(b)**, Cu-bpmpa **(d)**, and Cu-pmea **(f)**. A catalyst concentration of 0.3 mM was used for each complex. Conditions: pH 7 PB ($[PO_4]$ = 100 mM), 293 K, 50 mV s^{-1} scan rate, 0.196 cm^2 electrode surface area, 1600 RPM.

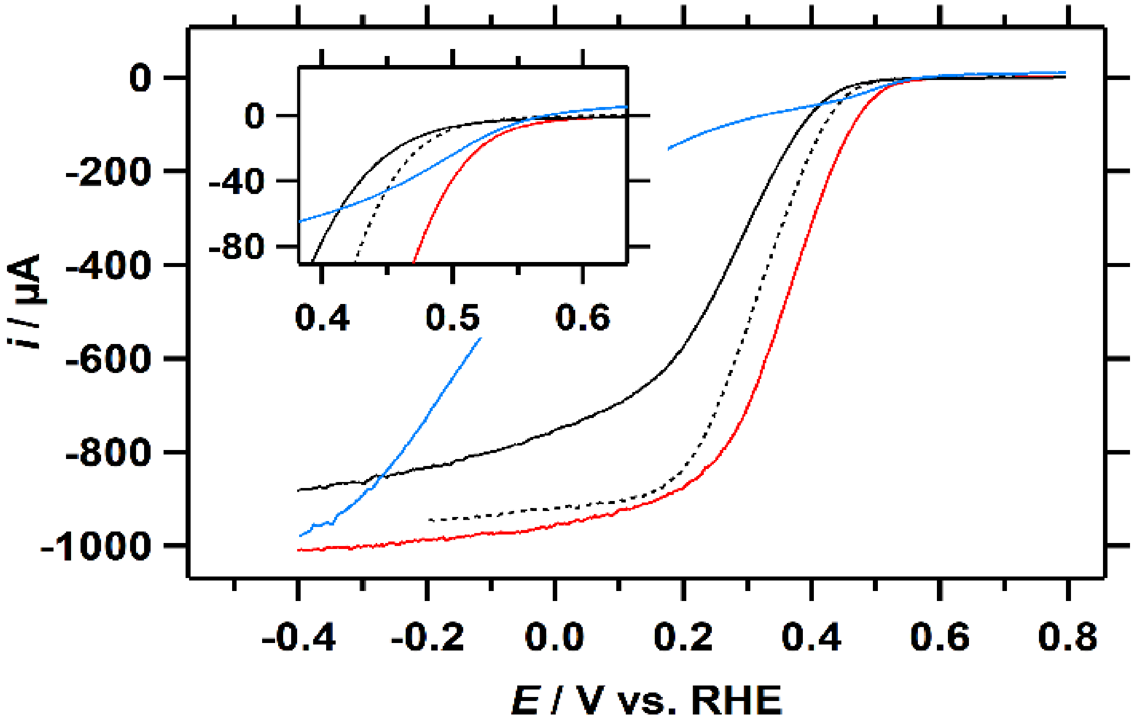

Figure S24 Onset potential for ORR in RDE experiments. RDE LSV experiments of Cu-fubmpa (black), Cu-pmea (red), Cu-bpmpa (blue), and Cu-tmpa (dotted line), under 1 atm O_2. LSV data for Cu-tmpa obtained from Ref 6. A catalyst concentration of 0.3 mM was used for each complex. Inset shows the onset of the catalytic waves. Conditions: pH 7 PB ($[PO_4]$ = 100 mM), 293 K, 50 mV s^{-1} scan rate, 0.196 cm^2 electrode surface area, 1600 RPM.

Table S4. Overview of the onset potentials determined for the ORR by Cu-fubmpa, Cu-pmea, and Cu-bpmpa in CV and RDE experiments, in a pH 7 phosphate buffer under 1 atm O_2.

Complex	*Average* $E_{\text{onset, ORR CV}}$ [b]	*Average* $E_{\text{onset, ORR RDE}}$ [b]
Cu-tmpa [a]	0.50	0.53
Cu-fubmpa	0.49 ± 0.006 (0.483, 0.499, 0.4753)	0.42 ± 0.015 (0.387, 0.429, 0.451)
Cu-pmea	0.50 ± 0.004 (0.507, 0.497)	0.56 ± 0.015 (0.571, 0.522, 0.580)
Cu-bpmpa	0.61 ± 0.0005 (0.607, 0.607, 0.605)	0.59 ± 0.004 (0.602, 0.585, 0.595)

[a] Determined from data from ref 6. Potentials are reported vs. RHE. [b] All onset potentials are determined as the average value of two or three measurements, individual values indicated in between brackets, in which the onset potential was determined as the potential at which the catalytic current becomes twice as large as the background current of the blank electrode. Conditions: pH 7 PB ([PO4] = 100 mM), 0.3 mM catalyst concentration, 293 K, 100 mV s^{-1} scan rate, 0.0707 cm^2 (CV) and 0.196 cm^2 (RDE) electrode surface area.

S8. H_2O_2 selectivity derived from RRDE measurements

In order to verify that all current recorded at the ring originates from oxidation of H_2O_2, RRDE LSV experiments were recorded for Cu-fubmpa, Cu-pmea, and Cu-bpmpa with the potential of the ring lowered from 1.2 V vs. RHE to 0.8 V vs. RHE (Figure S25). A potential of 0.8 V vs. RHE is high enough to oxidize all catalytic species but is below the oxidation potential of H_2O_2. As shown in Figure S25, the current collected at the ring belongs to the oxidation of H_2O_2 and confirms that all catalysts produce H_2O_2 during the ORR.

The selectivity of all catalysts during the ORR was determined using equation S3 and the data shown in the LSV experiments in Figure 7a (main text). In addition, the H_2O_2 selectivity of Cu-fubmpa, Cu-pmea, and Cu-bpmpa was determined at different potentials in CA experiments (Figure S26) and equation S3. To improve the accuracy of these measurements, the currents recorded at the ring during catalysis were corrected for the average currents measured with the disk set to 0.8 V vs. RHE during 60 seconds before the measurement. The final values of these CA measurements were determined as the average measured selectivity between 60-90 seconds of the experiment.

$$H_2O_2\,\% = \frac{2\frac{i_{ring}}{N}}{i_{disk}+\frac{i_{ring}}{N}}\, x\, 100\% \qquad \text{(S3)}$$

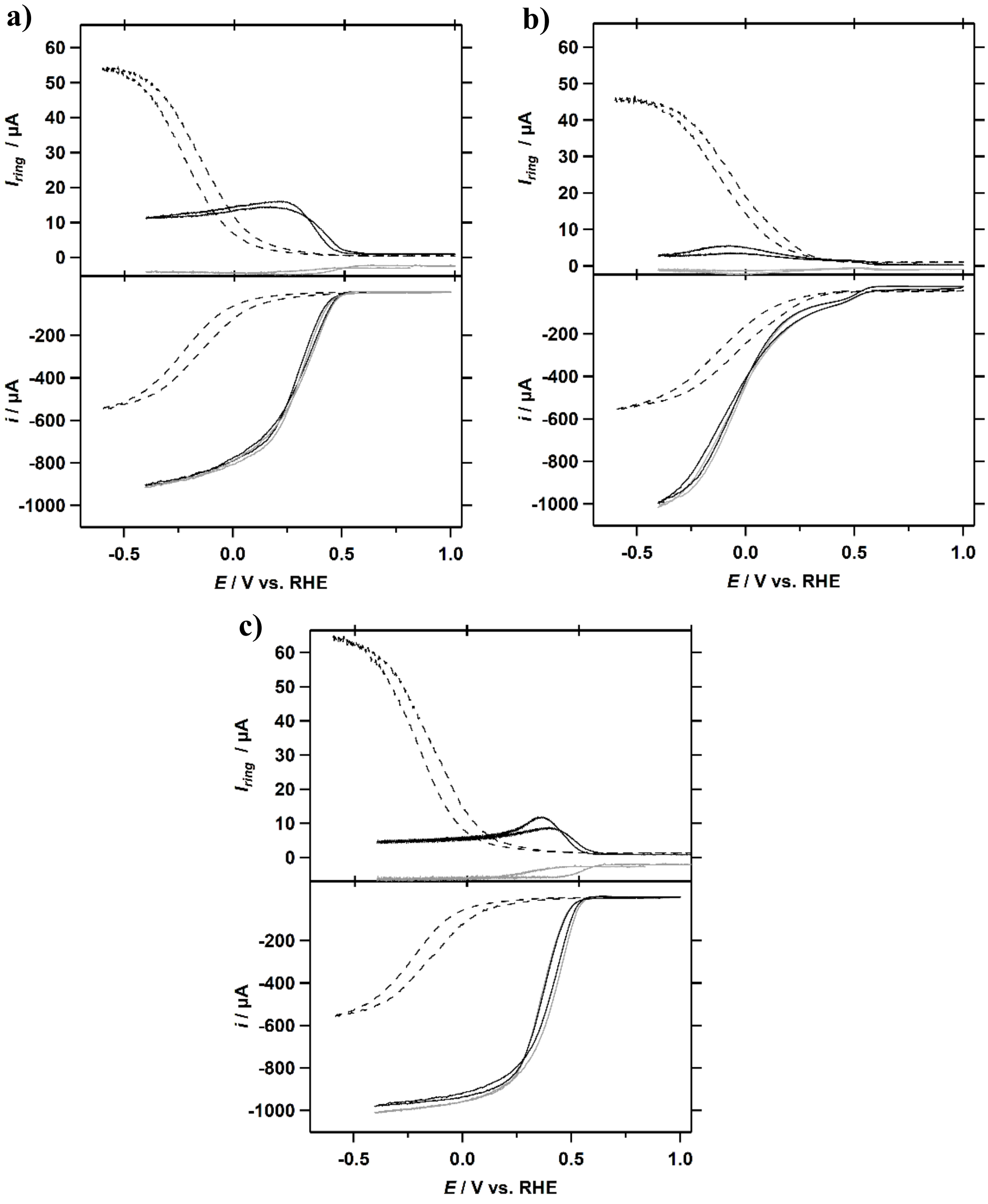

Figure S25. RRDE CVs of Cu-fubmpa **(a)**, Cu-bpmpa **(b)**, and Cu-pmea **(c)** under 1 atm O_2. All graphs show two separate measurements with the ring potential fixed at 1.2 V vs. RHE (black) and at 0.8 V vs. RHE (grey). CVs of the GC electrode in absence of catalyst shown for comparison (dashed line). A catalyst concentration of 0.3 mM was used for each complex. Conditions: pH 7 PB ($[PO_4]$ = 100 mM), 293 K, 50 mV s^{-1} scan rate, 0.196 cm^2 electrode surface area, 1600 RPM, Pt ring at 1.2 or 0.8 V vs. RHE.

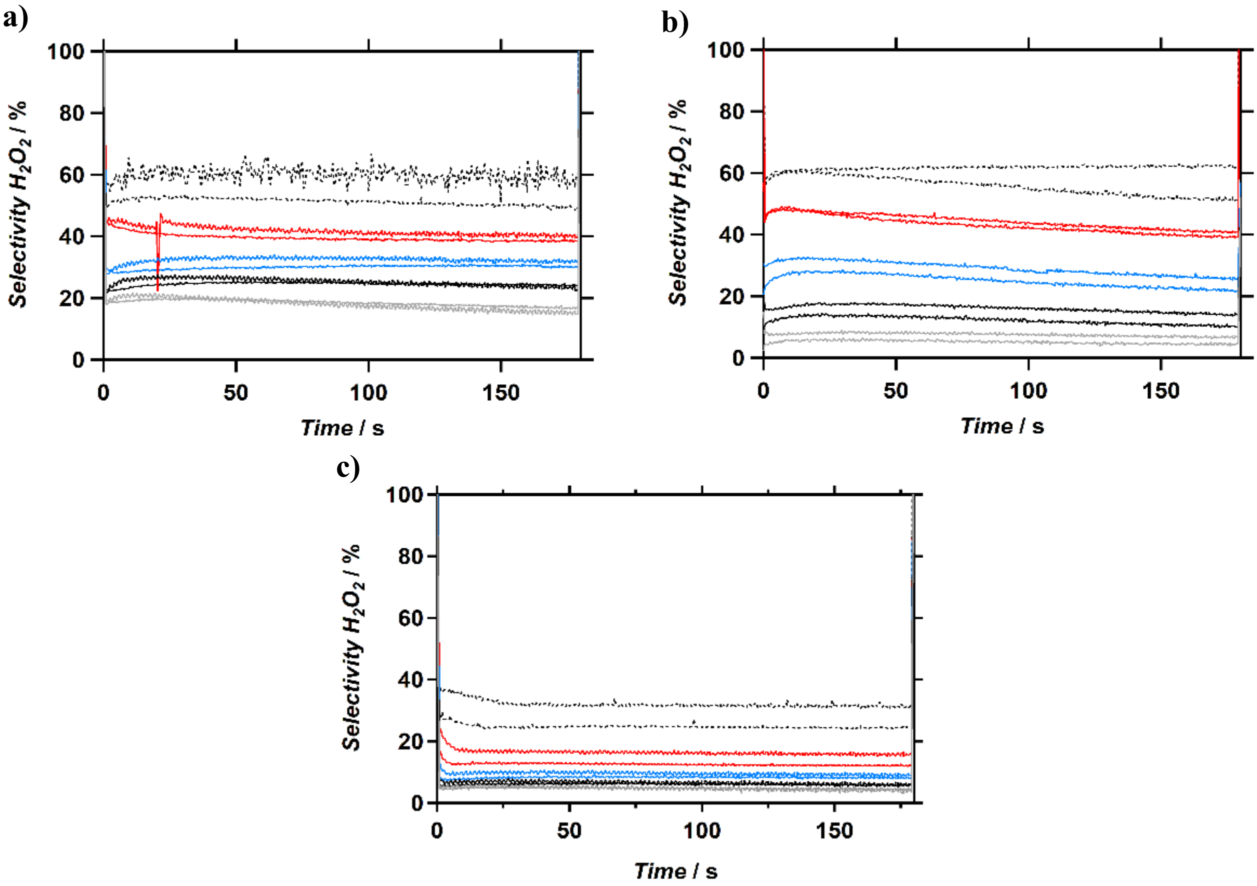

Figure S26. Selectivity of the ORR catalysed by Cu-fubmpa **(a)**, Cu-bpmpa **(b)**, and Cu-pmea **(c)** at 0.4 V (dotted line), 0.3 V (red), 0.2 V (blue), 0.1 V (black), and 0.0 V (grey) vs. RHE, obtained from RRDE experiments under 1 atm O_2. All measurements were repeated twice. A catalyst concentration of 0.3 mM was used for each complex. Conditions: pH 7 PB ($[PO_4]$ = 100 mM), 293 K, 0.196 cm^2 electrode surface area, 1600 RPM, Pt ring at 1.2 V vs. RHE.

S9. Computational methods

General method

DFT calculations and analysis of the calculated structures were performed using the Amsterdam Density Functional (ADF) engines of the AMS2022 program package developed by SCM.[10, 11] The B3LYP exchange correlation functional,[12-15] including Grimme's D4 dispersion correction,[16] was used for all DFT calculations. A triple zeta basis set with a polarization function (TZP)[17] was used. The COSMO implicit solvent model was used to account for solvent effects in water.[18] Starting geometries for Cu-tmpa, Cu-pmea, and Cu-bpmpa were obtained from reported coordinates of the crystal structures.[19-21] For Cu-fubmpa, the coordinates of the reported crystal structure in this study were used.

Binding energy calculations

For calculation of the binding energy of O_2 and H_2O_2 to all complexes, first a geometry optimization calculation of the Cu(II) state was performed based on the coordinates obtained from all crystal structures. In case of Cu-tmpa, Cu-bpmpa, and Cu-pmea the coordinating chloride atoms were replaced by a molecule of water and the new geometries, $[Cu(tmpa)H_2O]^{2+}$, $[Cu(bpmpa)H_2O]^{2+}$, and $[Cu(pmea)H_2O]^{2+}$, were optimized. Next, for Cu-tmpa and Cu-pmea the water molecule was removed to create a free coordination site for O_2 and the geometry of the +1 state of the complex was optimized to obtain the energy of the $[Cu(tmpa)]^{+}$ and $[Cu(pmea)]^{+}$ complexes (See Table S3 and S4). In case of fubmpa and bpmpa these are tridentate instead of tetradentate ligands, making it is unclear whether one or two H_2O molecules will leave upon reduction of the copper center. For this reason, energies were calculated for both $[Cu(fubmpa)]^{+}$, $[Cu(bpmpa)]^{+}$ as well as $[Cu(fubmpa)H_2O]^{+}$ and $[Cu(bpmpa)H_2O]^{+}$.

Next, a molecule of O_2 or H_2O_2 was added to all Cu(I) optimized geometries and the energy of the complexes was calculated from the optimized geometries. In case of O_2, both the triplet state and

broken-symmetry singlet state were obtained (See Table S5). In all cases, the triplet state corresponds to the ground state energy of the complexes, hence these structures were further analyzed. Comparing the O-O bond length of the obtained geometries to that of free O_2^- (1.364 Å) and O_2 (1.219 Å) verifies the formation of a superoxide complex upon binding of O_2 to copper (see Table S3). In case of H_2O_2, calculations of the triplet state showed that binding of H_2O_2 was energetically unfavored, hence the binding energy was calculated from closed-shell calculations. The geometric data in Table S4 shows that the M-O distance is large and H_2O_2 will only stay loosely bound to the metal center in this case, resulting in an O-O bond length that is close to the distance in the free H_2O_2 molecule (1.472 Å).

Next, the binding energies of O_2 and H_2O_2 were obtained using the following calculations:

Energy free O_2 molecule = -309.32 kcal/mol

Energy free H_2O_2 molecule = -526.97 kcal/mol

O_2 binding energy = [Energy Cu-O_2 complex] – [Energy Cu(I) complex] + 309.32 kcal/mol

H_2O_2 binding energy = [Energy Cu-O_2 complex] – [Energy Cu(I) complex] + 526.97 kcal/mol

Table S5. Overview of the calculated energies of geometry optimized structures to obtain the binding energy of O_2 and H_2O_2 to Cu-tmpa, Cu-pmea, Cu-fubmpa, Cu-bpmpa. All energies are given in kcal/mol.

Complex	Energy Cu(I) complex [a]	Energy Cu-O_2 complex[b]	Energy Cu-H_2O_2 complex [a]	Binding energy O_2	Binding energy H_2O_2
[Cu(tmpa)]$^+$	-6812.54	-7141.12	-7343.29	**-19.26**	**-3.78**
[Cu(fubmpa)]$^+$	-6430.65	-6753.56	-6961.8	**-13.59**	**-4.18**
[Cu(pmea)]$^+$	-7245.56	-7568.91	-7776.31	**-14.03**	**-3.78**
[Cu(bpmpa)]$^+$	-6385.6	-6702.88	-6917.97	**-7.96**	**-5.40**
[Cu(fubmpa)H_2O*]*$^+$	-6830.21	-7155.42	-7359.4	**-15.89**	**-2.22**
[Cu(bpmpa)H_2O*]*$^+$	-6786.38	-7106.17	-7317.06	**-10.47**	**-3.71**

[a] Closed shell calculation with charge +1. [b] Open shell triplet calculation with charge +1.

Table S6. Selected optimized geometrical parameters for Cu-tmpa, Cu-pmea, Cu-fubmpa, and Cu-bpmpa. All bond lengths are given in Ångstrom.

Complex	M-O distance Cu-O_2 complex [a]	O-O distance Cu-O_2 complex [a]	M-O distance Cu-H_2O_2 complex [b]	O-O distance Cu-H_2O_2 complex [b]
[Cu(tmpa)]$^+$	1.931	1.316	2.599	1.464
[Cu(fubmpa)]$^+$	1.942	1.321	2.923	1.460
[Cu(pmea)]$^+$	1.961	1.314	2.934	1.460
[Cu(bpmpa)]$^+$	1.953	1.312	2.614	1.460
[Cu(fubmpa) H_2O*]*$^+$	1.954	1.321	2.725	1.460
[Cu(bpmpa)H_2O*]*$^+$	1.962	1.313	2.614	1.458

[a] Open shell triplet calculation with charge +1. [b] Closed shell calculation with charge +1.

Table S7. Selected optimized parameters for Cu-tmpa, Cu-pmea, Cu-fubmpa, and Cu-bpmpa. All energies are given in kcal/mol.

Complex	Energy triplet [a]	Energy broken-symmetry singlet [b]
[Cu(tmpa)O$_2$]$^+$	-7141.12	-7138.54
[Cu(fubmpa)O$_2$]$^+$	-6753.56	-6751.31
[Cu(pmea)O$_2$]$^+$	-7568.91	-7566.32
[Cu(bpmpa)O$_2$]$^+$	-6702.88	-6700.13
[Cu(fubmpa) (H$_2$O)O$_2$]$^+$	-7155.42	-7153.2
[Cu(bpmpa)(H$_2$O)O$_2$]$^+$	-7106.17	-7103.49

[a] Open shell triplet calculation with charge +1. [b] Open shell calculation with charge +1.

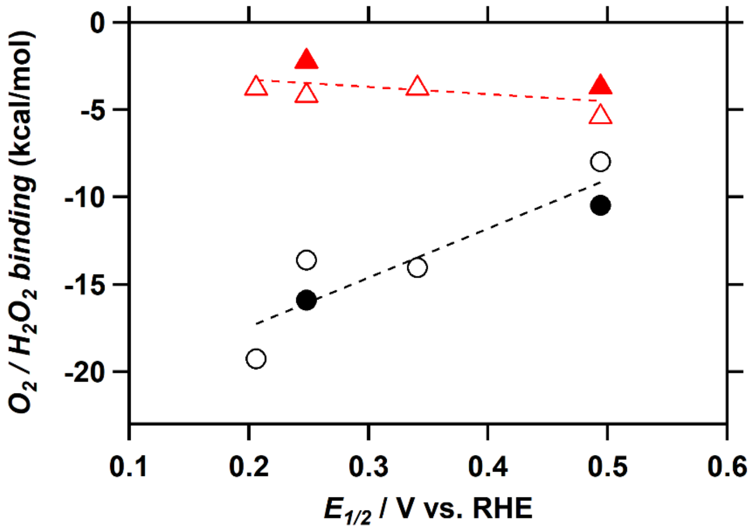

Figure S27. Plot of the calculated binding energies of O_2 (black circles) and H2O2 (red triangles) to the Cu(I) state of $[Cu(tmpa)]^+$ ($E_{1/2}$ = 0.206), $[Cu(fubmpa)]^+$ ($E_{1/2}$ = 0.248), $[Cu(fubmpa)H_2O]^+$ ($E_{1/2}$ = 0.248), $[Cu(pmea)]^+$, $[Cu(bpmpa)]^+$ ($E_{1/2}$ = 0.494), and $[Cu(bpmpa)H_2O]^+$ ($E_{1/2}$ = 0.494) versus the $E_{1/2}$ of the respective catalysts. For Cu-fubmpa and Cu-bpmpa the two data points represent the complexes with (solid circle/triangle) and without (open circle/triangle) a molecule of water coordinated to the copper site. The linear fit was fitted through the data points that represent the complexes without additional water (open), R^2 = 0.84 for ORR and R^2 = 0.72 for HPRR.

S10. References

1. Foxon, S. P.; Walter, O.; Schindler, S., Syntheses and characterization of copper(II) complexes of the new Ligands N-[(2-pyridyl)methyl]-2,2 '-dipyridylamine and N-[Bis(2-pyridyl)methyl]-2-pyridylamine. *Eur. J. Inorg. Chem.* **2002,** (1), 111-121.
2. Lonnon, D. G.; Craig, D. C.; Colbran, S. B., Rhodium, palladium and platinum complexes of tris(pyridylalkyl)amine and tris(benzimidazolylmethyl)amine N_4-tripodal ligands. *Dalton Trans.* **2006,** (31), 3785-97.
3. Sheldrick, G. M., SHELXT - Integrated space-group and crystal-structure determination. *Acta Crystallogr. A* **2015,** *71*, 3-8.
4. Nicholson, R. S.; Shain, I., Theory of Stationary Electrode Polarography - Single Scan + Cyclic Methods Applied to Reversible Irreversible + Kinetic Systems. *Anal. Chem.* **1964,** *36* (4), 706-&.
5. Bullock, R. M.; Appel, A. M.; Helm, M. L., Production of hydrogen by electrocatalysis: making the H-H bond by combining protons and hydrides. *Chem. Commun.* **2014,** *50* (24), 3125-3143.
6. Langerman, M.; Hetterscheid, D. G. H., Fast Oxygen Reduction Catalyzed by a Copper(II) Tris(2-pyridylmethyl)amine Complex through a Stepwise Mechanism. *Angew. Chem. Int., Edit.* **2019,** *58* (37), 12974-12978.
7. Langerman, M.; Hetterscheid, D. G. H., Mechanistic Study of the Activation and the Electrocatalytic Reduction of Hydrogen Peroxide by Cu-tmpa in Neutral Aqueous Solution. *Chemelectrochem* **2021,** *8* (15), 2783-2791.
8. Smits, N. W. G.; van Dijk, B.; de Bruin, I.; Groeneveld, S. L. T.; Siegler, M. A.; Hetterscheid, D. G. H., Influence of Ligand Denticity and Flexibility on the Molecular Copper Mediated Oxygen Reduction Reaction. *Inorg. Chem.* **2020,** *59* (22), 16398-16409.
9. Smits, N. W. G.; Rademaker, D.; Konovalov, A. I.; Siegler, M. A.; Hetterscheid, D. G. H., Influence of the spatial distribution of copper sites on the selectivity of the oxygen reduction reaction. *Dalton Trans.* **2022,** *51* (3), 1206-1215.
10. te Velde, G.; Bickelhaupt, F. M.; Baerends, E. J.; Fonseca Guerra, C.; van Gisbergen, S. J. A.; Snijders, J. G.; Ziegler, T., Chemistry with ADF. *J. Comput. Chem.* **2001,** *22* (9), 931-967.
11. AMS 2022.1 SCM. Rüger, R.; Franchini, M.; Trnka, T.; Yakovlev, A.; van Lenthe, E.; Philipsen, P.; van Vuren, T.; Klumpers, B.; Soini, T., Eds. http://www.scm.com: Theoretical Chemistry, Vrije Universiteit, Amsterdam, The Netherlands.
12. Vosko, S. H.; Wilk, L.; Nusair, M., Accurate spin-dependent electron liquid correlation energies for local spin density calculations: a critical analysis. *Can. J. Phys.* **1980,** *58*, 1200-1211.
13. Becke, A. D., Density-functional thermochemistry. III. The role of exact exchange. *J. Chem. Phys.* **1993,** *98* (7), 5648-5652.
14. Stephens, P. J.; Devlin, F. J.; Chabalowski, C. F.; Frisch, M. J., Ab Initio Calculation of Vibrational Absorption and Circular Dichroism Spectra Using Density Functional Force Fields. *J. Phys. Chem.* **1994,** *98* (45), 11623-11627.
15. Lee, C.; Yang, W.; Parr, R. G., Development of the Colle-Salvetti correlation-energy formula into a functional of the electron density. *Phys. Rev. B* **1988,** *37* (2), 785-789.
16. Caldeweyher, E.; Ehlert, S.; Hansen, A.; Neugebauer, H.; Spicher, S.; Bannwarth, C.; Grimme, S., A generally applicable atomic-charge dependent London dispersion correction. *J. Chem. Phys.* **2019,** *150* (15), 154122.

17. Van Lenthe, E.; Baerends, E. J., Optimized Slater-type basis sets for the elements 1-118. *J. Comput. Chem.* **2003,** *24* (9), 1142-56.

18. Pye, C. C.; Ziegler, T., An implementation of the conductor-like screening model of solvation within the Amsterdam density functional package. *Theor. Chem. Acc.* **1999,** *101*, 396-408.

19. Karlin, K. D.; Hayes, J. C.; Juen, S.; Hutchinson, J. P.; Zubieta, J., Tetragonal vs. Trigonal Coordination in Copper(II) Complexes with Tripod Ligands: Structures and Properties of $[Cu(C_{21}H_{24}N_4)Cl]PF_6$ and $[Cu(C_{18}H_{18}N_4)Cl]PF_6$. *Inorg. Chem. 21* (11), 4106-4108.

20. Schatz, M.; Becker, M.; Thaler, M.; Hampel, F.; Schindler, S.; Jacobson, R. R.; Tyeklar, Z.; Murthy, N. N.; Ghosh, P.; Chen, Q.; Zubieta, J.; Karlin, K. D., Copper(I) Complexes, Copper(I)/O_2 Reactivity, and Copper(II) Complex Adducts, with a Series of Tetradentate Tripyridylalkylamine Tripodal Ligands. *Inorg. Chem.* **2001,** *40*, 2312-2322.

21. Foxon, Simon P.; Walter, O.; Schindler, S., Syntheses and Characterization of Copper(II) Complexes of the New LigandsN-[(2-Pyridyl)methyl]-2,2′-dipyridylamine andN-[Bis(2-pyridyl)methyl]-2-pyridylamine. *Eur. J. of Inorg. Chem.* **2002,** *2002* (1), 111-121.
